# Molecular guidance cues in the development of visual pathway

**DOI:** 10.1007/s13238-017-0490-7

**Published:** 2017-11-27

**Authors:** Yupu Diao, Yuqing Chen, Peijun Zhang, Liyuan Cui, Jiayi Zhang

**Affiliations:** 1 Institutes of Brain Science, State Key Laboratory of Medical Neurobiology and Collaborative Innovation Center for Brain Science, Fudan University, Shanghai, 200032 China; 2 Department of Ophthalmology, Eye and ENT Hospital, Fudan University, Shanghai, 200031 China

**Keywords:** molecular guidance cue, optic chiasm, dLGN, thalamocortical axons, corticothalamic projection

## Abstract

70%–80% of our sensory input comes from vision. Light hit the retina at the back of our eyes and the visual information is relayed into the dorsal lateral geniculate nuclei (dLGN) and primary visual cortex (V1) thereafter, constituting the image-forming visual circuit. Molecular cues are one of the key factors to guide the wiring and refinement of the image-forming visual circuit during pre- and post-embryonic stages. Distinct molecular cues are involved in different developmental stages and nucleus, suggesting diverse guidance mechanisms. In this review, we summarize molecular guidance cues throughout the image-forming visual circuit, including chiasm determination, eye-specific segregation and refinement in the dLGN, and at last the reciprocal connections between the dLGN and V1.

## Introduction

Neuronal development and axonal wiring in mice visual system rely on many factors such as molecular guidance cues, retinal waves and visual experience. Retinal waves are the driving force in visual pathway patterning, especially during the first two postnatal weeks. Disrupting retinal waves in stage II and stage III respectively cause severe impairment in the eye-specific segregation and retinotopic refinement in the dLGN (Rossi et al., 2001; Muir-Robinson et al., 2002). In ferret, blocking stage II retinal waves damages the ocular dominance column segregation (Huberman et al., 2006). Meanwhile, blockage of visual experience by dark-rearing and binocular deprivation disrupts the maturation of the orientation selectivity in ferret (Chapman and Stryker, 1993; White et al., 2001). Compared with retinal waves and visual experience, molecular guidance cues function earlier because the guidance cues exist as early as E13 when the first axons arrive at the optic chiasm (O’Leary et al., 1983). To some degree, molecular guidance cues provide the basic framework for visual circuit wiring and refinement (McLaughlin and O’Leary, 2005). The wiring of the image-forming visual circuit is consisted of several essential stages including optical chiasm fate determination, eye-specific segregation in the dLGN, precise wiring of the axonal circuitry within the dLGN, thalamocortical connection establishment and corticothalamic feedback innervations. In this review, we collect and summarize the guidance molecules that are reported to be of great importance to the development of essential stages along the retina-dLGN-V1 pathway.

## Molecular guidance cues at the optic chiasm

**Figure 1:**
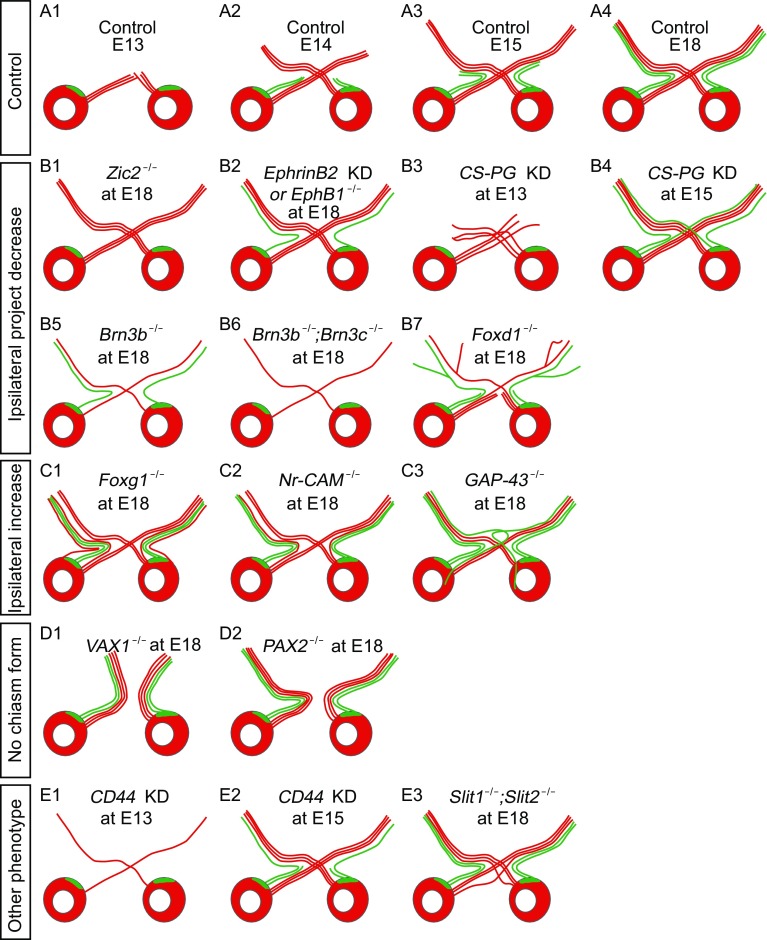
**Schematics of genes and molecules involved in the pathfinding of RGCs axon at the optic chiasm**. (A1–5) Normal development of RGCs axon pathfinding at the optic chiasm. (B1–7) Molecules lead to ipsilateral projection reduction at the optic chiasm in mice. (C1–3) Increase of ipsilateral projections at the optic chiasm. (D1–2) No optic chiasm formation in the VAX1 and PAX2 knockout mice. (E1–3) Some other phenotypes observed at the optic chiasm in different mice models

Axons of retinal ganglion cells (RGCs) exit the eyeball, form the optic tract and arrive at the optic chiasm, a point where RGC axons from the two eyes encounter and face the choice of crossing (contralateral projection) or uncrossing (ipsilateral projection). Within one retina, about 97%–98% RGCs project contralaterally and 2%–3% RGCs project ipsilaterally (Drager and Olsen, 1980). RGCs are generated at about E11.5 and the first contralateral-projecting RGC axons arrive at the optic chiasm at around E13. Axons of a subtype RGCs develop into permanent ipsilateral projections at around E14 (O’Leary et al., 1983; Guillery et al., 1995). In the following day, ipsilateral projections arrive at the optic chiasm (Fig. 1A1–4). Ipsilateral-projecting RGCs arise exclusively from ventrotemporally (VT) retina, but contralateral-projecting RGCs are distributed throughout the whole retina. In the early studies, it is believed that positional cues in the retina are not sufficient to determine the crossing or uncrossing at the optic chiasm (Colello and Guillery, 1990). Subsequent studies show that the pathfinding of ipsilateral and contralateral RGC axons are determined by molecular cues but not the competition between binocular axons (Wang et al., 1995; Xiang et al.). Dozens of molecular guidance cues are precisely expressed in spatial and temporal patterns coordinately at the optical chiasm or in the RGCs, controlling crossing or uncrossing of RGC axons (Nakagawa et al., 2000; Leung et al., 2004; Grinberg and Millen, 2005). Such molecules have been intensively studied in the past two decades using knock-down or knock-out mice. Several well-defined phenotypes are observed in these mutant mice, which could be divided into four groups: 1. ipsilateral projection reduce in *Zic2*, *EphB1*/*epherin-B2*, *CS-PG*, *Brn3b* ^−/−^, *Brn3b* ^−/−^; *Brn3c* ^−/−^ and *Foxd1* null mice (Fig. 1B1–7); 2. ipsilateral projection increase in *Foxg1*, *Nr-CAM*, *GAP-43* and *Isl2* eliminating mice (Fig. 1C1–3); 3. no optic chiasm formation in *Vax1* and *Pax2* knockout mice (Fig. 1D1–D2); 4. other molecule guidance cues show interesting and dynamic phenotypes, such as *CD44* loss of function mice display opposite phenotypes at different development stages and *Slit1*/*2* knockout mice exhibit two chiasms (Fig. 1E1–3).

### Genes related to ipsilateral projection reduction

Zinc finger transcription factor, *Zic2*, is an important factor in neuronal development. In human beings, diseases including neural tube defects and holoprosencephaly are closely related to the abnormal expression or missing of *Zic* gene (Grinberg and Millen, 2005). However, knocking out *Zic2* in mouse is lethal during perinatal period or shortly after birth (Nagai et al., 2000). Among the three homologies in mouse, *Zic2* exclusively expressed at ventrotemporal peripheral retina. Besides, the expression of *Zic2* is spatially and temporally aligned with the outgrowth of ipsilateral projections during E14.5–E17.5. Ipsilateral projections are reduced dramatically in *Zic2* knock-down mice compared with control and heterozygous mice. Over-expression of *Zic2 in vitro* leads to increased neurite growth and ejection at the optic chiasm. The loss- and gain-of function together demonstrate that *Zic2* is necessary and sufficient to determine ipsilateral projecting RGCs (Herrera et al., 2003).

Previous study demonstrates that EphB1/epherin-B2 is functional in controlling ipsilateral projections at the optic chiasm in mammals (Nakagawa et al., 2000). Further studies show that EphB1 expressed restrictedly in ventrotemporal RGCs and epherin-B2 located specifically in the chiasm midline. Surprisingly, both EphB1 and epherin-B2 express in a similar parabola pattern with a peak at E15.5, a time point when the most ipsilateral projections are generated. The expression level is lower at E14.5 and E17, which are two time points related to the start and the end of ipsilateral projection development (Drager, 1985; Rachel et al., 2002). Further studies show that blocking epherin-B2 *in vitro* and knocking out *EphB1* gene in mice both result in remarkable reduction of ipsilateral projections from the retina (Williams et al., 2003). Gain-of-function in EphB1 via *in utero* retinal electroporation method leads to a dramatic increase in ipsilateral projections by converting some of the contralateral-projecting axons to ipsilateral-projecting ones (Petros et al., 2009). These results indicate that epherin-B2 expression in the midline is repulsive for ipsilaterally projecting EphB1-positive axons from ventrotemporal retina, mediating the divergence of retinal axons at the optic chiasm (Williams et al., 2003).

Aggrecan, versican, phosphacan and neurocan are members of the chondroitin sulfate proteoglycan (CS-PG) family. Their expressions are concentrated in the retina or optic chiasm (Bandtlow and Zimmermann, 2000; Leung et al., 2004; Popp et al., 2004). The expression of these molecules has spatial and temporal patterned during RGC axon pathfinding at the optic chiasm (Leung et al., 2004). Extracted CS-PG has been verified to function as an inhibitory factor or repellent molecule during outgrowth of RGC axons *in vitro* by time-lapse microscopy (Snow et al., 1991). Similarly, after digestion of chondroitin sulfate moieties from CS-PG molecules at the optic chiasm using chondroitinase ABC enzyme at E13, contralateral projecting axons are disorganized pre-midline and post-midline. Removal of CS-PG at E14 and E15 produces remarkable reduction of ipsilateral projections while contralateral projections are not affected. Besides, after treatment with enzyme, the size of the growth cone increases both before and after they cross the midline at E13–15 (Chung et al., 2000).

Brn3 POU-domain transcription factors (*Brn3b* and *Brn3c*) are detected in projecting neurons including RGCs (Gan et al., 1999; Erkman et al., 2000). The first detection of Brn3b in mice RGCs was at E11.5 (Xiang et al., 1995). In *Brn3b* ^−/−^ single mutant mice, a lot of RGCs are missing in the retina, and projections at optic chiasm are reduced and misled towards the hypothalamus (Erkman et al., 2000). *Brn3b* ^−/−^;*Brn3c* ^−/−^ double knockout mice display even greater loss of RGCs and defect in axonal outgrowth than *Brn3b* ^−/−^ single mutant mice (Wang et al., 2002a). One raised question here is whether the abnormal/reduced projection at the chiasm is caused by the severe loss of retinal ganglion cells in the *Brn3b* ^−/−^;*Brn3c* ^−/−^ and *Brn3b* ^−/−^ mice. Explant of *Brn3c* containing retina in *Brn3b* ^−/−^ mutant mice has remarkable recovery in the neurite outgrowth, demonstrating its growth promoting function. Furthermore, in *Brn3b* ^−/−^ mice, the morphology of outgrowth axon shows abnormal scattering and avoiding going through the optic disk. These results together illustrate the growing guidance function of these cues (Wang et al., 2002a). Hence, the missing of ipsilateral projections in *Brn3b* ^−/−^;*Brn3c* ^−/−^ double knockout mice partially results from a serious loss of RGCs in the ventral-temporal retina, possibly also because of the loss of growth promoting and guidance from *Brn3b* and *Brn3c* molecules.

Winged helix transcription factor (*Foxd1*), also known as brain factor 2 (BF-2), is a powerful molecule in binocular visual system establishment that guides VT RGC specification, optic chiasm and retinotopic formation (Herrera et al., 2004; Carreres et al., 2011). *Foxd1* mRNA can be detected in the retina at E11–E17 with a higher expression level in the peripheral VT quadrant at E12–E14, which decreases at E17 (Marcus et al., 1999). In *Foxd1* ^−/−^ null mutant, a majority of axons terminate at the chiasm and the remaining axons further separate into four branches after leaving the chiasm with altered ratio of ipsilateral and contralateral projections (Herrera et al., 2004). These studies suggest that *Foxd1* is required in determining the crossing of RGC axons. Some important guidance molecules such as *Foxg1*, *Zic2* and *EphB1* also dramatically alter their expression patterns in *Foxd1* ^−/−^ null mutant (Herrera et al., 2004). Loss of inhibitory force imposed by *Foxd1* to *Foxg1* directly causes the expansion of *Foxg1* and *Slit2* expression into the territory where *Foxd1* expresses. The most surprising results in *Foxd1* knockout mice is that a major loss of *Zic2* and EphB1 in ventrotemporal retina still results in numerous ipsilaterally projecting RGCs. *Foxd1* deletion also perturbs the expression of topographic mapping effectors such as EphA6/ephrinA5. Hence, the complex phenotype at the optic chiasm in *Foxd1* ^−/−^ null mutant may due to a combination of numerous ectopically expressed molecular cues including *Foxd1*.

### Genes related to ipsilateral projection increase

Another member of winged helix transcription factor family is *Foxg1*, which is also called brain factor 1 (BF-1). This gene expresses in the nasal retina and optic chiasm, whose territory is complementary to *Foxd1* (Xuan et al., 1995; Marcus et al., 1999). In *Foxg1* null mutant mice, there is eight times increase in ipsilateral projections without altered expression patterns of uncrossing related transcription factors Nkx2.2 and cell surface molecule SSEA-1 or ephrin B2 (Pratt et al., 2004). The primary study demonstrated reduced proliferation and immature differentiation in a group of cells in the telencephalon in *Foxg1* null mutant mice (Xuan et al., 1995). Pratt and colleagues speculated that *Foxg1* was not autonomous required by the retina (Pratt et al., 2004). Another phenotype of the mutant mice includes eye malformation (Xuan et al., 1995; Pratt et al., 2004).

Cell adhesion molecule (Nr-CAM) expresses in dorsal-temporal retina (contralateral projecting region) at E12.5, reaching a peak at E15 throughout the whole retina and radial glia cells of chiasm midline, followed by a gradual decrease at the chiasm midline until E18.5 with a sharp decrease in the peripheral retina. Detailed studies on the axons show the highest expression level at the end of the axons (Lustig et al., 2001; Williams et al., 2006). Notably, the spatial distribution of *Nr-CAM* expression is complementary to *Zic2* and opposite to *EphB1* (Herrera et al., 2003). *In vitro* blocking of Nr-CAM by antibody increases axons that do not cross the midline (Lustig et al., 1999; Lustig et al., 2001; Williams et al., 2006). Results in the homozygous *Nr-CAM* knockout mice display a dramatically larger size in ipsilateral projections at late embryonic stage, as well as substantially reduced number of contralateral projections (Sakurai et al., 2001; Williams et al., 2006). Further quantification of ipsilateral and contralateral cells in the retina under retrograde labeling experiment shows a smaller proportion of ipsilateral projections at E15.5–E17.5, but a higher fraction of ipsilateral projections at E18.5, suggesting *Nr-CAM* is a late-stage contralateral-specific cue (Williams et al., 2006). As we described previously in this review, *EphB1* ^−/−^ null mutant showed a remarkable reduction in ipsilateral projections (Williams et al., 2003). *Nr-CAM* ^−/−^;*EphB1* ^−/−^ double knockout mice exhibit a larger size of ipsilateral projections (Williams et al., 2006).

GAP-43 is a membrane protein highly expressed in the growth cone (Skene, 1990). In *GAP-43* ^−/−^ mutant mice, both the contralateral and ipsilateral projecting RGCs are distributed all over the retina (Sretavan and Kruger, 1998), whereas in control mice, the ipsilateral projecting RGCs locate exclusively in the ventrotemporal part of the retina. However, the morphology of the eye and retina is normal (Kruger et al., 1998). Dye labeling illustrates an un-parallel growth of RGCs axons before crossing the chiasm. When coming to the chiasm, the RGC axons cannot cross the lateral wall and hence the ipsilateral and contralateral projections grow in a random manner (Strittmatter et al., 1995; Sretavan and Kruger, 1998). *GAP-43* ^−/−^ mutant mice have a comparable number of ipsilaterally and contralaterally projecting neurons growing in semicircular trajectories. More surprisingly, the ipsilaterally projecting axons lack a sharp turn to the designed track and re-cross after its first crossing at the chiasm, which results in a 4-day delay in reaching the optic tract on both sides (Kruger et al., 1998; Sretavan and Kruger, 1998).

Besides, the knockout of *Isl2* can also lead to a major increase in ipsilateral projections at the chiasm as well as the dLGN, which will be discussed in detail in the dLGN session.

### No optic chiasm formation

*Vax1* or *Pax2* knockout mice have no optic chiasm formation with all RGC axons projecting ipsilaterally and coloboma eyes with unsealed retina. Vax1 is secreted from the ventral hypothalamus and diffuses to the RGC axons, playing an essential role in the RGC axonal growth at the chiasm (Kim et al., 2014). In *Vax1* ^−/−^ mutant mice, the RGC axons do not enter the brain but stall outside the presumptive chiasm (Bertuzzi et al., 1999). One possible mechanism is that the expression of attractive factor Netrin is reduced or lost along the RGC axon pathway to chiasm, producing overwhelming repulsive forces on contralateral projections. By contrast, RGC axons in *Pax2* null knockout mice project ipsilaterally only, due to the loss of activity gene *sonic-hedgehog* (*shh*) expression gap along the A-P axis where the contralateral projections go (Torres et al., 1996). Consequently, neither *Vax1* nor *Pax2* is a path-defining gene. Instead, they can prohibit the formation of the optic chiasm.

### Other molecule guidance cues

CD44 is a transmembrane glycoprotein which is highly expressed in neurons of ventral diencephalon from E13 (Sretavan et al., 1994). The co-labelling with β III tubulin reveals that CD44 exists in the early-born cells of the optic chiasm. *In vitro* antibody-blocking experiment shows a dynamic change of CD44 function. Briefly, antibodies blocking the function of CD44 cause significant reduction of contralateral projection axons, leaving the ipsilateral projection axons unchanged in E13 and E14. However, the situation reverses at E15, when disrupting the function of CD44 at the chiasm sharply reduced ipsilateral projections but the contralateral projections are normal (Lin and Chan, 2003).

Slit1 and Slit2 are conserved protein among species and first demonstrated to function during the commissural axon pathway-finding in *Drosophila* (Rothberg et al., 1990). Slit/Robo acts as repellent in many axon-wiring processes including the crossing at the optic chiasm (Erskine et al., 2000; Niclou et al., 2000). In *Slit1* ^−/−^ or *Slit2* ^−/−^ single mutant, no significant defect at the optic chiasm is found. However, in *Slit1* ^−/−^;*Slit2* ^−/−^ double mutant, the most obvious defect is that another commissure appears which lies anterior and ventral to the normal one. Some of the pioneer axons leave the main axon bundle and project to the pre-chiasm area, forming a second chiasm-like commissure (Plump et al., 2002). Slit protein has the same role in both mice and *Drosophila*, which is a midline repellent during development of the neuron system (Herrera et al., 2004; Long et al., 2004).

**Figure 2:**
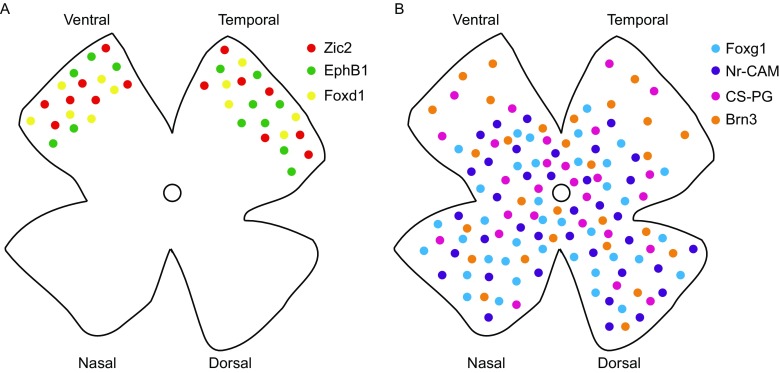
**Spatial distribution of the guidance cues in the retina**. (A) Molecules expressed in the VT region. (B) Molecules expressed across the whole retina or the complementary territory

**Figure 3:**
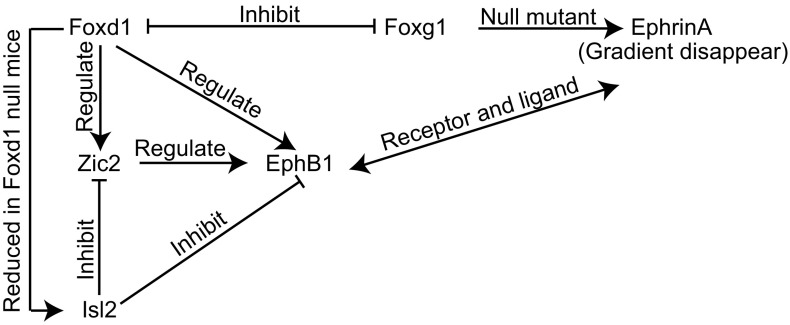
**Network of genes and molecules during the pathfinding of RGC axons at the optic chiasm**

In summary, RGC axons in a variety of genetic mice models display distinct and independent phenotypes on the crossing or uncrossing at the chiasm. There are several possible mechanisms: 1). Molecules that guide the retinal axons growth from the retina to chiasm are expressed in spatially distributed mode in the retina (Fig. 2). 2). We have summarized a network of genes and molecules that could be involved collectively in axon guidance at the optic chiasm (Fig. 3). *Foxd1* and *Foxg1* inhibit each other and guide the retinal axons through different pathways. *Foxg1* is important for maintaining the ephrinA gradient. The ephrinA and EphB1 are ligand and receptor that repel each other at the chiasm. At the same time, *Foxd1* regulates the expression of *EphB1*, *Zic2* and *Isl2*. *Zic2* also regulates the expression of *EphB1*. In *Foxd1* ^−/−^ mutant, the expression of *Isl2* reduces dramatically and *Isl2* inhibits *EphB1* and *Zic2*. These molecules together modulate the axon growth from the retina to optic chiasm.

## Genes involved in the development of eye-specific segregation in the dlgn

**Figure 4:**
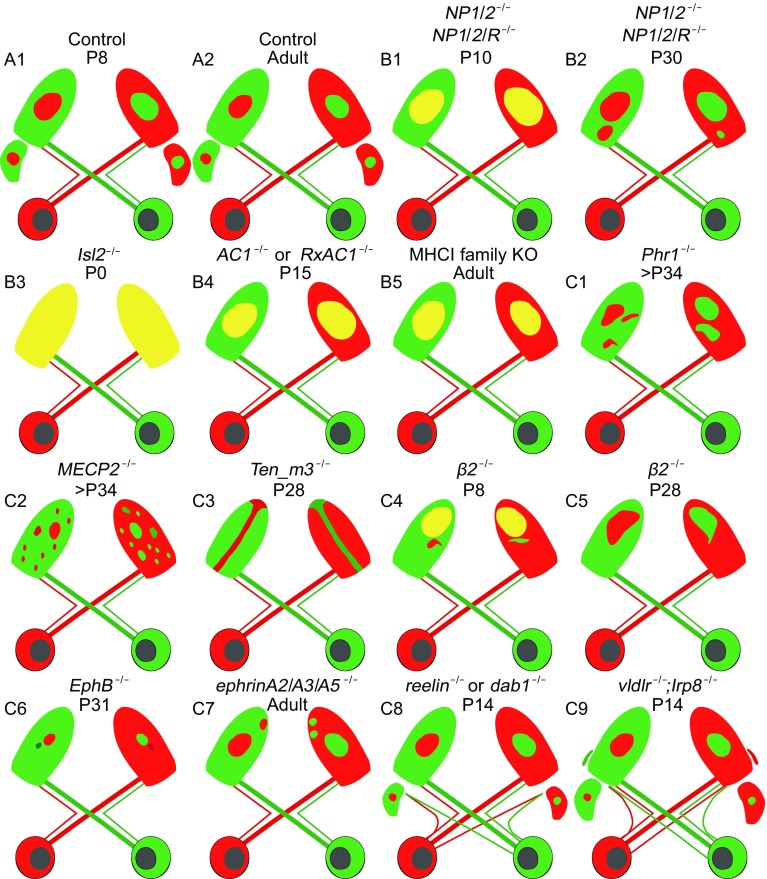
**Schematics of genes and molecules involved in the retinal axon targeting and eye-specific segregation in the dLGN**. (A1–2) Development of RGC axons targeting in the dLGN in control mice. (B1–5) Mice with increased overlap projections in the dLGN. (C1–9) Abnormal ipsilateral projection in the dLGN

In mice, all RGC axons go through the optic chiasm and enter several thalamic nuclei, among which a large fraction of axons target and arborize in the dLGN (Morin and Studholme, 2014). In the dLGN, axons from the two eyes are well separated into two distinct territories, which underlies the binocular vision (Huberman et al., 2008). During the development of eye-specific segregation in the dLGN, axons from the two eyes first mingle together, and later on separate and refine into their target territory. RGC axons go through a complicated process in innervating the dLGN and forming synaptic connections with cells in the dLGN. Molecular guidance activities during this process are of great importance. In this article, we summarized most of the known guidance molecules in the dLGN. Defects in the eye-specific segregation in the dLGN can roughly be divided into two groups: increase in overlapping projections (projections from both contralateral and ipsilateral eyes) and disorder in ipsilateral projection patterns (Fig. 4).

### Increased ipsilateral/contralateral overlap projections to the dLGN

Neuronal pentraxins (NPs) family members NP1, NP2 and NP receptor (NPR) play a role in synaptic formation (Reid and Blobel, 1994; Schlimgen et al., 1995; Dodds et al., 1997). There are obvious immunohistochemistry signals in the dLGN with spatial-temporal gradient. At P7, NP1 is immuno-active in cells throughout the dLGN and NP2 mostly located at the inner core of the dLGN. At P14, NP1 expression is lower than that at P7, and NP2 shows denser expression in the outer shell layer of the dLGN (Bjartmar et al., 2006). In *NP1*/*2* null mutant mice, there is no significant difference between *NP1*/*2* null mutant and wild type mice at P4. By P10, *NP1*/*2* null mutant mice have an obvious defect in eye-specific segregation and refinement that the contralateral axons occupy the whole territory and the ipsilateral/overlap projection areas are significantly larger. Up to P30, eye-specific segregation and refinement is improved compared with that at P10, but ipsilateral projections still occupy a larger domain (Bjartmar et al., 2006; Koch and Ullian, 2010). *NP1*/*2*/*NPR* triple or single knock mice have no worse phenotype, demonstrating that *NP1* together with *NP2* are required for eye-specific segregation and refinement in dLGN. Further study in the retina illustrates a delayed functional maturation of glutamatergic synapses (Koch and Ullian, 2010). Whether the NPs affect eye-specific segregation and refinement through disrupting the retinal waves or other guidance molecule is still unknown.

*Isl2*, LIM-homeodomain transcription factor, is expressed exclusively and strongly in contralaterally projecting RGCs throughout the retina with a decrease of expression density in the ventral-temporal crescent where all ipsilateral projections originate (Pak et al., 2004). Knock out of this gene causes upregulation of *Zic2* and *EphB1* as well as increase in *Zic2*-positive RGC numbers. In *Isl2* double knockout mice (Thaler et al., 2004), ipsilateral projections to the dLGN significantly increase, which coincide with an increase in *Zic2* positive RGC cells (Pak et al., 2004). In *Isl2* null mutant, the ipsilateral projections filled almost the entire dLGN. Despite a significant increase in the ipsilateral projections, the contralateral projections remain unchanged. Studies in the Zebrafish, in which all RGCs project contralaterally in the optic chiasm, show ipsilateral projections in the *Isl2* mutant. These results also indicate an autonomous function of Islr2 during the axon path-finding (Panza et al., 2015). These results together demonstrate a specific role for *Isl2* in inhibiting the ipsilateral projection. Here *Isl2* exhibits almost the same function in inhibiting the ipsilateral projections to the tectum and dLGN in mice and zebrafish, respectively (Thaler et al., 2004; Panza et al., 2015). Because the *Isl2* null mutant pups died within 24 h after birth, the function of *Isl2* during the eye-specific refinement is unknown.

Adenylate cyclase1 (AC1) catalyzes to produce cAMP in a Ca^2+^/calmodulin-dependent manner, and is required for the establishment of eye-specific segregation (Dhande et al., 2012). Deletion of *AC1* in mice results in abnormal retinogeniculate projections, but the retinal waves are normal (Plas et al., 2004; Nicol et al., 2006a). Study with single RGC axon labeling demonstrates continuous increase in the size and decrease in the density of axonal arborization between P4 and P16 (Dhande et al., 2012). Furthermore, *RxAC1* KO mice in which *AC1* is conditionally knocked out in the retina also have larger ipsilateral targeted zones in the dLGN and a significant decrease in eye-specific segregation, but these phenotypes are not as severe as that in *AC1* ^−/−^ null mouse (Swindell et al., 2006). Nevertheless, knock out *AC1* gene in the superior colliculus (SC) display no abnormality in eye-specific segregation (Dhande et al., 2012). These results confirm that the abnormal retinogeniculate projection is partially due to pre-synaptic *AC* deletion. *In vitro* study reveals that AC1 is involved in the Ephrin-A5 dependent retinal axon retraction, suggesting the potential mechanism for the enlarged targeting zone in *AC1* ^−/−^ null mouse (Nicol et al., 2006b).

Major histocompatibility complex class I (MHCI) is comprised of a large number of molecules (>50) which have important roles in the immune system (Zinkernagel and Doherty, 1979). Until now, a lot of studies illustrate vital roles of MHCI in central nervous system development (Syken et al., 2006; International Schizophrenia et al., 2009; Shatz, 2009). Class I MHC mediate signaling pathway, including H2-K^b^ and H2-D^b^ as two members belonging to class Iα subfamily, is involved in retinogeniculate projections; β2M, TAP1, C1q and C3 co-subunits are necessary for normal and functional expression of MHCI at the cell-surface; CD3ζ and PirB are components of two distinguished MHCI receptors that participate in this signaling pathway. Each of these genes expresses in specific neuronal cell types, except for H2-K^b^, H2-D^b^ and C1q that are co-localized in the terminals of retinogeniculate projections axons (Huh et al., 2000). Several knock out mouse lines including *H2-K* ^*b−*/−^;*D* ^*b−*/−^, *β2M* ^−/−^; *TAP1* ^−/−^, *β2M* ^−/−^, *CD3ζ* ^−/−^, *C1q* ^−/−^, *C3* ^−/−^ show disrupted eye-specific segregation with similar phenotype (Huh et al., 2000; Datwani et al., 2009). At P5, there is no significant difference between control and knock out mice, but by P10, a time window for retinogeniculate projection refinement, the ipsilateral projections of knockout mice are abnormally larger compared with the control ones, leading to an increase in the overlap fractions, and this phenotype persists until P30 (Datwani et al., 2009). C1q and C3 have been identified to function in synaptic elimination without disturbing patterns of retinal inputs (Stevens et al., 2007). This phenotype may partially explain the unusually large territory occupied by the ipsilateral projections, and may further provide support for the enhancement of ocular dominance plasticity exhibited in *H2-K* ^b−/−^;*D* ^b−/−^ mice, which is not a topic of this article. It is still unclear whether the mechanisms for these similar phenotypes are the same or not.

### Factors contributing to the abnormal ipsilateral projections in the dLGN

*Phr1* is known as a regulator of synapse formation and has been intensively studied in *Drosophila*, *C*. *elegant* and *Zebrafish* (Schaefer et al., 2000; DiAntonio et al., 2001; Bloom et al., 2007). This gene is highly expressed in the retina but it turns out that the development of the retina is not severely affected after conditionally knocking out *phr1* gene in the retina. Moreover, retinal waves and EphA expression are not disturbed (Pfeiffenberger et al., 2005; Culican et al., 2009). However, it does affect the eye-specific segregation in the dLGN after different cre-line driven knocking out, such as *Pax*-Cre and *Math*5-Cre. The ipsilateral and contralateral projections can be segregated, but there are several more patches of ipsilateral projections in the ventral-medial area of dLGN (Culican et al., 2009). Quantification of ipsilateral projections between different mutants and control mice reveals comparable proportions of ipsilateral projections. Further, the morphology of the axon growth cones is abnormal *in vitro* culture (Burgess et al., 2004; Lewcock et al., 2007). These results indicate a retinal-independent mechanism for *phr1* gene in guiding eye-specific segregation in the dLGN. This gene is particularly interesting because it functions differ in different animal models. In *Drosophila* and *C*. *elegant*, this gene functions as synaptic synaptogenesis, growth and termination effector (Schaefer et al., 2000; DiAntonio et al., 2001). In zebrafish, *Phr1* affects the optic tectum projection (D’Souza et al., 2005). In mice, *phr1* knockout mice has abornmal eye-specific segregation (Culican et al., 2009).

Methyl-CpG-binding protein 2 (MECP2), a transcriptional regulator, possesses evident role during synaptic development (Lewis et al., 1992; Fukuda et al., 2005; Chao et al., 2007). In *MECP2* null mice, an abnormally increased innervation of retinal axons to dLGN is evident. By P27-34, the ipsilateral patch is less condensed than control and presents an irregular edge, but there is no significant difference between them. Up to P46-51, the ipsilateral projection zone stretches throughout the dLGN that results in an increase in overlap fraction (Noutel et al., 2011). However, vesicular glutamate transporter 2 (VGLUT2), a transmitter at the retinogeniculate projection terminal in the dLGN, are reduced in both density and size from P30 to P60 (Schafer et al., 2016). This time-dependent phenotype may reflect the synaptic maintenance function of *MECP2* at late stage of development.

Ten-m3, a highly conserved type II transmembrane protein, expresses in high-ventral to low-dorsal gradient in the retina and this gradient remains in its corresponding retinotopic recipient in dLGN, exerting an important role in eye-specific targeting (Oohashi et al., 1999). Indeed, high-throughput screening in mice indicates that this gene is specifically expressed in visual projecting neurons (Leamey et al., 2008). Tracing retinogeniculate projections in *Ten-m3* loss function mutant mice displays complicated and seriously disordered ipsilateral mapping in different parts of the dLGN. At the caudal region of the dLGN, ipsilateral projections form several patches. An elongated phenotype manifests in the middle region and a narrow stripe across the dLGN along the dorsal-medial and ventral-lateral axis in the rostral region. The majority area occupied by the contralateral patch is unchanged. Moreover, the distribution of contralateral and ipsilateral projecting RGCs is undisturbed by *Ten-m3* knockout, as well as the optic chiasm (Leamey et al., 2007). The irregular retinogeniculate mapping has passed on to the visual cortex and further affects some of the visual-related behavior, especially in the vertical placement test and visual cliff test (Leamey et al., 2007). Overexpression of *Ten-m3* in the retina leads to a noticeable increase in ipsilateral projections in wallaby model (Carr et al., 2014). These results confirm that Ten-m3 work as a molecular guidance cue in the eye-specific mapping.

β2 is a subunit of the neuronal nAChR (Xu et al., 1999) that mediates ACh-transduced waves between P1 and P7. In *β2* ^−/−^ knockout mice, the unusual eye-specific segregation is temporally dynamic. At P8, contralateral projections occupy the whole dLGN including the area that generally receives ipsilateral projections. The ipsilateral projections still arrive at the dorsal area but form a larger zone, making them segregated poorly from the contralateral projections and resulting in an increase in overlap fraction than wild type. Up to P14, the segregation becomes much better than that at P8. However, the ipsilateral projection does not form a clear dense patch and exhibits an irregular shape. At P28, axonal segregation from ipsi- and contra- eye improves comparing to P14, but still with an irregular territory of the ipsilateral projections (Rossi et al., 2001; Muir-Robinson et al., 2002).

The Eph family tyrosine kinase receptors and their cell surface-binding ligands ephrins serve as a concentration-dependent cue for the retinotopic mapping of RGCs to the dLGN and SC (Gebhardt et al., 2012; Cang and Feldheim, 2013). However, Eph subfamily knockout mice display different phenotypes. Take *EphB1* null mice and *ephrinA2*/*A3*/*A5* triple knockout mice for instance. In *EphB1* ^−/−^ mice, as summarized in chiasm guidance session, the ipsilateral projections are visibly reduced. Further observations show that, consistent with the phenotype at the optic chiasm, there is evident shrinkage in the ipsilateral projection patch than control mice. Besides, a surprisingly dense contralateral RGC axon patch is also observed adjacent to the ipsilateral area. VT retina tracing (compared with whole retina/eye tracing) finds out that the ectopic contralateral axon patch originates from VT RGCs (Rebsam et al., 2009). EphB1 is not expressed in the dLGN, so the defect most probably roots in the retina or chiasm. In contrast to receptor EphB, ephrinA2/A3/A5 ligand expresses in the developing thalamus. EphrinA2 and ephrinA5 express in the same gradient, but ephrinA3 express only in a few cells of the dLGN (Pfeiffenberger et al., 2005). In *ephrinA2*/*A3*/*A5* triple knockout mice, the ipsilateral projection separates into several patches with one in the destination and the others lie at the medial edge of the dLGN. Though several patches are observed, the ratio of overlap and ipsilateral projection is not clearly changed compared to the control ones. Further study in retina waves displays the relatively normal activity pattern (Pfeiffenberger et al., 2005).

Reelin is a secreted extracellular glycoprotein with N-terminal f-spondin domain and C-terminal rich in positive charged amino acid group (D’Arcangelo et al., 1995; D’Arcangelo et al., 1997). The genomic organization of Reelin is highly similar between mice and human, implying evolutionary conservation in the gene structure (Royaux et al., 1997). This gene came into sight when it was discovered to function in the synaptic development of neurons such as plasticity, polarization and targeting (Borrell et al., 1999; Matsuki et al., 2010; Rogers et al., 2011). In a chip screening study aimed at capturing differentially expressing genes between dLGN and vLGN, *reelin* gene is identified to involve in nuclei-specific axon guidance. Reelin binds to very-low-density lipoproteins receptor (VLDLR) and low-density lipoproteins receptor-related protein 8 (LRP8) *in vivo*. After binding to these receptors, Reelin activates disabled-1 (Dab1) to function downstream (Howell et al., 1997; Sheldon et al., 1997; Trommsdorff et al., 1999). In *reln* ^−/−^ mutant, two striking defects are found at P1. First, there is a gap between IGL and the medial-lateral part of vLGN, which may be caused by the reduced projection from RGCs, and this reduction in RGC innervation leads to the shrink in the vLGN territory; another abnormality is a large number of misrouted axons originating from both eyes’ intrinsically photosensitive retinal ganglion cell (ipRGCs) at the vLGN, but axons aiming at the dLGN is unaffected (Su et al., 2011). This confirms the function of reelin in specific targeting at vLGN and IGL but not dLGN. Reduced targeting in IGL and misrouted fibers also exist in 50% *Dab1* mutant at a less modest degree compared to *reln* ^−/−^ mice, while in the other half, only misrouted axons are found, again less than those in *reln* ^−/−^ mutant.

*vldlr* ^−/−^ and *lrp8* ^−/−^ single mutant mice display no defects, while double mutant mice show more severe phenotype than *reln* ^−/−^ null mutant (See above), providing evidence for compensatory functions between the two receptors. In *vldlr* ^−/−^;*lrp8* ^−/−^ double mutant mice, two distinct abnormalities emerge, which are not seen in any of the *reln* ^−/−^ or *dab* ^−/−^ mutants. Specifically, in *vldlr* ^−/−^;*lrp8* ^−/−^ mutant, an additional patch from contralateral eye lies lateral-dorsal just under and adjacent to optic tract bunch. Besides, an ectopic patch comprising of ipsilateral and contralateral RGCs projections exist at the dorsal-medial head of the dLGN. Moreover, mice carrying one copy of the two receptors including *vldlr* ^−/−^;*lrp8* ^+/−^ and *vldlr* ^+/−^;*lrp8* ^−/−^ display similar defects but not so serious as those in double null mutants (Su et al., 2013). Further study interestingly demonstrates that these dorsal-medial projections are the results of mispositioned IGL neurons. Results verify that Reelin is also expressed in this region, overlapping with misrouted arbors (Su et al., 2011). The mechanism of why IGL neurons migrate to the dorsal-medial head of the dLGN is unclear.

In addition to the molecular guidance cues that affect the eye-specific segregation in the dLGN, a kind of ipRGCs also plays a role in the retinal axon targeting. *Opn4* ^*DTA*/*DTA*^ ipRGC ablation mice demonstrate a significant increase in the overlap projections to the dLGN, leaving the ipsilateral and contralateral projections similar to those in the wild-type mice. Moreover, the territories of the ipsilateral projection form irregular patches. These phenotypes occurred as early as P8 and last into adulthood. Further studies in the ipRGC ablation mice show altered retina activity pattern at P6. These results indicate that the abnormal retinal projections in the ipRGC ablation mice may be partially due to the abnormal retinal activity in early postnatal days (Chew et al., 2017).

## Molecules as guidance cues on the thalamocortical pathway

**Figure 5:**
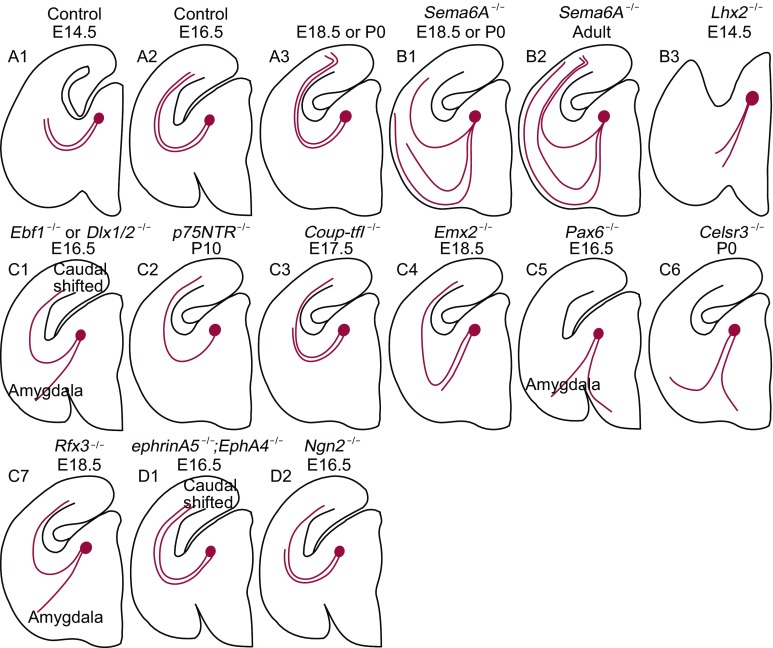
**Schematics of the pathfinding of thalamocortical axons after knocking out genes related to guidance cues**. (A) Normal development of thalamocortical axon pathfinding from E14.5 to P0. (B1–3) Phenotypes of thalamocortical axons after knocking out TC axon guidance cues including *Semaphorin-6A* and *Lhx2*. (C1–7) Phenotypes of thalamocortical axons after knocking out TC pathway intrinsic cues including *Ebf1*, *Dlx1/2*, *p75NTR*, *Coup-tfI*, *Emx2*, *Pax6*, *Celsr3* and *Rfx3*. (D1–2) Phenotypes of thalamocortical axons after knockout of topographic mapping cues such as *ephrin-A5*; *EphA4* and *Ngn2*. Red dots represent dLGN projecting neurons. Red lines stand for TC axons

Thalamocortical (TC) connection plays a great role in conveying visual information to the neocortex. During development, TC axons initiate from dLGN nucleus, pass through prethalamus, and turn laterally into the ventral telencephalon. They subsequently approach the internal capsule and finally project to the neocortex. The establishment of TC connection involves a dynamic interplay between molecular guidance cues in the thalamic axons and intrinsic cues along the pathway. A large number of molecules defined as pathfinding guidance cues have been investigated in transgenic mice. Here, we summarize these molecular cues and divide them into three main groups. The first group is related to the TC axon guidance including *Semaphorin-6A*, *Lhx2* (Fig. 5A1–B3); the second is the intrinsic cues along the TC pathway including *Ebf1*, *Dlx1*/*2*, *p75NTR*, *Coup-tfI*, *Emx2*, *Tbr1*, *Gbx2*, *Pax6, Celsr3* and *Rfx3* (Fig. 5C1–7); the third group is topographic mapping cues including Eph/ephrin and *Ngn2* (Fig. 5D1–2).

### TC axon guidance cues

*Semaphorin-6A* is widely and intensively expressed in the dorsal thalamus, the amygdala and the ventral telencephalon, with a relatively lower expression level in the cortex during the embryonic stage. In functional *Semaphorin-6A* lacking mutants, the experiments of placing DiI in the dLGN, together with neurofilament (NF) immunohistochemistry and placental alkaline phosphatase (PLAP) staining, show a large portion of labeled TC axons misrouted at the ventral telencephalon and the amygdala, with only a small amount of neurons reaching the cortex at E16.5-P0. Interestingly, retrograde labeling shows that the presumptive back tracing target the dorsal thalamus is replaced by the lateral ventrobasal. Meanwhile, the histology of the cortex is unchanged, as well as the retina projections which also have a large amount of Semaphorin-6A expression. However, in the early postnatal stage, many of the cortex-projecting dLGN axons follow a different route, which is probably due to their mispositioning at the embryonic stage (Little et al., 2009). Among these axons arriving at the visual cortex, some of them are misrouted to the superficial layer of the cortex. These results indicate the important function of guidance of Semaphorin-6A during the TC axons pathfinding at the embryonic and early postnatal stages.

*Lhx2* transcription factor belongs to the LIM-homeodomain family of transcription factors that is expressed in the embryonic dorsal thalamus and participates in regulating the dorsal thalamus patterning (Nakagawa and O’Leary, 2001). In *Lhx2* deletion mutant embryos, thalamic axons are not able to enter the ventral telencephalon and aberrant topography exists *in vitro*. In absence of *Lhx2*, a number of dorsal-thalamus-specific markers or patterning-related molecules are normally expressed, implying the normal development of dorsal thalamus nucleus. Further study finds that internal capsule (IC) cells are greatly reduced in *Lhx2* mutants, which is a putative explanation for the misrouted TC axons. Besides, a repellent cue Sema6A is verified to be up-regulated in the ventral telencephalon, which may take part in the abnormal sorting of TC axon bundles (Lakhina et al., 2007). Thus, the disruptions of TC pathfinding and topographic projection are not caused by the cell-autonomous role of Lhx2 in the thalamus, but the impairment in the ventral telencephalon.

### The intrinsic cues along the TC pathway

In the following part we will introduce the intrinsic cues in different nuclei along the TC pathway including the ventral telencephalon (*Ebf1*, *Dlx1*/*2*), the subplate neurons (*p75NTR*, *Coup-tfI)* and the cortex (*Emx2)*. “Handshake” modal cues *Tbr1*, *Gbx2* are described in detail in **“Handshake” modal cues** session, as well as some other genes such as *Pax6*, Celsr3 and Rfx3.

A number of genes such as *Ebf1* and *Dlx1*/*2* express mainly in the ventral telencephalon, which play crucial roles in directing TC pathfinding. Early B-cell factor, Ebf1, also named *Olf-1*, *O*/*E-1*, *COE1*, encodes a transcription factor verified to function in the development of basal ganglia (Hagman et al., 1993; Garel et al., 1999; Dubois and Vincent, 2001). Ebf1 expresses in the dorsal thalamus and basal ganglia along the thalamocortical pathway at E14.5. In *Ebf1* ^−/−^ mutant embryos, axons from the dLGN are misrouted inside the basal ganglia towards the amygdala. Additionally, the projections between thalamic nuclei and neocortical domains have a caudally shifted topography in the neocortex. Further study finds that a group of cells in the basal ganglia express *Sema6a* at a lower level than control (Garel et al., 2002). These phenotypes lead to the conjecture that structural defects in the basal ganglia can affect the thalamocortical pathfinding. Another mutant described below supports this hypothesis to some degree. *Dlx1* and *Dlx2* encode homeodomain transcription factors, which are known to express in the basal ganglia. *Dlx1*/*2* ^−/−^ embryos exhibit severe stagnation in the differentiation of basal ganglia cells (Bulfone et al., 1993; Anderson et al., 1997). In *Dlx1*/*2* ^−/−^ embryos, some thalamic axons fail to grow past the basal ganglia. Similar as *Ebf1* ^−/−^ mice, a few axons finally find ways into the cortex, but topographic mapping has an obvious caudal shift (Garel et al., 2002).

p75 neurotrophin receptor (*p75NTR*) expresses in the subplate neurons and exhibits a low-rostral to high-caudal gradient from E14.5 to P7, a time window when the thalamic axons invade into the cortex. *p75NTR* is also highly expressed in the posterior thalamus at E14.5. In mice lacking *p75NTR*, innervation of visual cortex from the dLGN is half gone compared with the wild type mice. This deficiency is observed in both P10 and adult mice by dye retrograde and radiation labeling respectively at different ages. Moreover, the study of placing DiI crystal at the LGN shows that TC axons follow the normal pathway through the internal capsule and get close to the subplate of the neocortex, but stop there. Lack of *p75NTR* does not affect the programed cell death in subplate neurons. In addition, axonal innervation of the auditory and somatosensory cortex in *p75NTR* knockout mice is normal. Hence, *p75NTR* plays a great role in the thalamocortical innervation to cortex through an undefined mechanism (McQuillen et al., 2002).

Chicken ovalbumin upstream promoter-transcription factors *Coup-tfI* is reported to be of great importance for neuron differentiation and development of the central neural system (Qiu et al., 1997b; Yamaguchi et al., 2004). In *Coup-tfI* mutant mice, the majority of TC axons do not pass through the internal capsule, while a few axons that reach the subplate fail to innervate the proper layer. Besides the defect in thalamocortical innervation, the layer IV cortical neurons go through massive cell death during the development, resulting in a loss of layer IV (Zhou et al., 1999). Another study shows high expression of calretinin protein in subplate neurons (Fonseca et al., 1995), a calcium-binding protein that should not be detected in subplate neurons at E14.5 and E16.5, implying some bugs in the differentiation of these neurons during this period. Hence, the possible mechanism regarding defect in *Coup-tfI* null mutant mice is that in absence of the guidance of *Coup-tfI*, thalamocortical neurons get lost in the internal capsule, and few survivors come to the subplate region, but encounter the poorly differentiated subplate neurons and at last stop there at P0, which may be responsible for the loss of layer IV neurons.

Mouse transcription factor Emx2, a member of the empty spiracles family, shows a rostral-caudal and medial-lateral gradient expression in the dorsal part of developing cerebral cortex, including neuroepithelium, ventricular zone and Cajal-Retzius cells during embryonic stages (Gulisano et al., 1996; Bishop et al., 2000; Cecchi, 2002). It is known that *Emx2* gene is critical to the cortical patterning and area specification (Mallamaci et al., 2000). In *Emx2* ^−/−^ embryos, thalamocortical projections separate into two branches, with some of them end at the border of telencephalon and diencephalon, and the others grow through the telencephalon-diencephalon border into the designed path to the cortex (Lopez-Bendito et al., 2002). Histochemistry study provides evidence that there is an enlarged cell-free area in the internal capsule where presumably the abnormal thalamocortical pathway arises, which may explain the thalamocortical projection defect in *Emx2* ^−/−^ mice (Lopez-Bendito et al., 2002).

“Handshake” modal cues *Tbr1* and *Gbx2* are described in detail in **“Handshake” modal cues** session.

There are many other genes determining thalamocortical and corticothalamic projections at the same time, for instance, *Pax6*, *Celsr3* and *Rfx3*. The transcription factor *Pax6* expresses in both the cortex and thalamus. In *Pax6* ^−/−^ mutants, thalamocortical axons are remarkably reduced at E14.5 as they grow into the subpallium. Dye tracing experiments find that no axons innervate the cortex. The corticothalamic axons do not reach their target either (Hevner et al., 2002).

Protocadherin *Celsr3* expresses in the developing brain and is crucial for the development of central neuron bundles (Formstone and Little, 2001; Tissir et al., 2002). In *Celsr3* mutant mice, histology staining shows malformation or absence of the internal capsule. DiI labeling exhibits disordered connections between the thalamus and the cortex. At around E14.5, axons from the thalamus pass through the diencephalon ventrally in a clear bundle. The axons then separate into two groups, with one group going towards the dorsal base of the brain and the other turning externally through the basal forebrain and heading to the homolateral cortical marginal zone at P0, leading to aberrant corticothalamic axon projections (Tissir et al., 2005).

Ciliogenic transcription factor (*Rfx3*) is involved with ciliogenesis and cilia function. In the *Rfx3* ^−/−^ mutant, some thalamocortical axons fail to grow out of the diencephalon and abnormally project towards the amygdala, while corticothalamic axons abnormally migrate towards the pial surface of the ventral telencephalon (Magnani et al., 2015).

### The topographic cues

In the complex process of thalamocortical projection establishment, a precise topographic pattern between thalamus nuclei and a specific cortical area is extremely crucial. It’s well documented that projections from thalamic nuclei into the cortex is topographically organized along rostrocaudal and mediolateral cortical axes (Lopez-Bendito and Molnar, 2003). However, the underlying mechanism of the exquisite thalamocortical topographic mapping remains poorly understood. The ventral telencephalon is thought to be an “intermediate target”, responsible for early sorting of thalamocortical projections before the tract innervates the cortex (Lakhina et al., 2007).

Ephrins and their receptors, Eph/ephrin signaling pathway, function in the cell-cell interactions and guidance of axon growth corns in the developing neocortex (Egea and Klein, 2007). As thalamocortical axons extend towards the ventral telencephalon, Eph/ephrin plays a crucial role in the early topographic sorting and determination of the topographic mapping of TC axons after their innervation into the cortex. Mice deficient in EphA4, EphA7, or both ephrin-A5 and EphA4, display a fully penetrant topographic caudal shift of TC axons (Dufour et al., 2003).

The bHLH transcription factor *Ngn2* expresses spatially and temporally in the dorsal thalamus at the embryonic stage. In *Ngn2* knockout mice, there is a pronounced pathfinding defect in the ventral telencephalon that axons from the rostral dorsal thalamus shift caudally and appear in the internal capsule as opposed to the normal medial-lateral projections in normal mice (Seibt et al., 2003). In conclusion, *Ngn2* plays an autonomous role in the topographic organization of TC projections.

## Functions of genes involved in the corticogeniculate feedback projections

**Figure 6:**
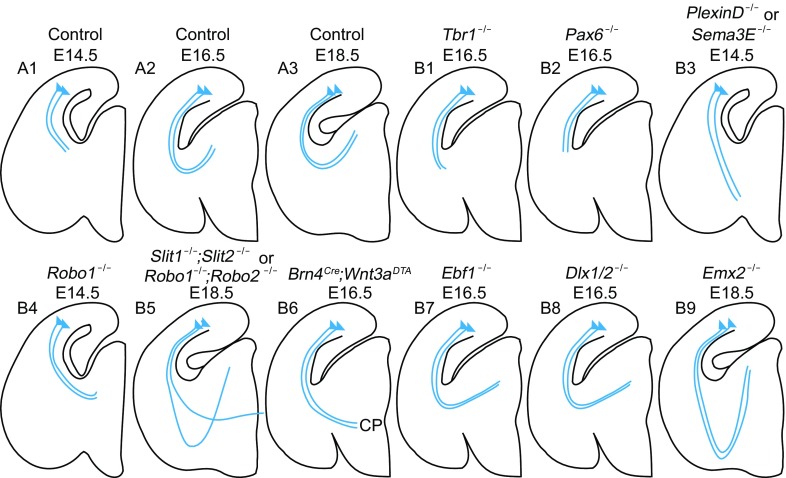
**Schematics of defects in corticothalamic axons pathfinding**. (A1–3) Normal developmental of corticothalamic axons pathfinding. (B1–9) Misled corticothalamic axons after knock out of some closely related cues. These cues are listed along the paths of corticothalamic axons. Blue lines represent CT axons. Blue triangles represent corticothalmic projecting neurons

Robust and specific connections between the cortex and dLGN are established during the late embryonic stage or shortly after birth and cortical axons stay outside of the dLGN for several days before innervation (Brooks et al., 2013). Corticogeniculate (CT) projecting neurons generates from the embryonic subplate and identify by specific transcription factors. The corticothalamic axons pass by several nuclei, with one day’s pause during E13.5–E14.5 and a sharp turn for axons to target the dLGN (Fig. 6A1–3). A lot of molecular guidance cues attend this process, ensuring precise pathway finding and proper targeting (Fig. 6B1–9).

### Fate determination and outgrowth of the neocortex

*Fezf2*, *Ctip2* and *Tbr1* are three known genes express in designated layers or positions where they interact with each other and control different corticofugal projections, such as corticothalamic, corticospinal (CS) (Su et al., 2011) and subcerebral projections (Srinivasan et al., 2012). Among these three genes, T-box brain gene, *Tbr1* is certified as one of the main factors to determine the corticothalamic projection neurons in the neocortex. First, *Tbr1* is highly expressed in preplate glutamatergic neurons during the embryonic stage (Bulfone et al., 1999) in the subplate and postnatal (P0.5) layer VI, and the high expression level lasts until adulthood (Hevner et al., 2001). Second, DiI tracing and PLAP labeling both show that corticothalamic fibers fail to target the dLGN, in *Tbr1* knock out (*Tbr1* ^−/−^) mice, implying the defect of corticothalamic projections (Hevner et al., 2001). Third, the ectopic expression of *Tbr1* in layer V leads to abnormal projections to the thalamus (McKenna et al., 2011), indicating its important role in the initial fate determination of cortical neurons. After the classification by different transcription factors, Tbr1-expressing neurons start to stretch their axons downwards at about E10 under the control of semaphorins family that act bi-functionally and in gradient. Expressed in the cortical plate (CP) to repel CT axons, Sema3A combines with attraction forces imposed by Sema3C in the intermediate zone (IZ) which is in a lateral to medial gradient, and together they induce a tendency of lateral growth of CT axons (Leyva-Diaz and Lopez-Bendito, 2013). Notably, metalloproteinases (MMP3) participates in this course due to its ability to activate the signaling transduction of Sema3A and Sema3C by cleavage (Gonthier et al., 2007). In IZ zone, CT axons encounter further attractive and repulsive cues induced by SemaE and SemaD respectively. The expression territory of SemaE and SemaD overlap spatially in IZ, with the attractive cue being stronger (Bagnard et al., 1998). We believe that the initially unbalanced forces of attraction and repellent guide the cortical axons to move further ventrally and laterally. Meanwhile, Sema3A and Sema5B in the ventricular zone (VZ) exert repulsive functions on outgrowing axons, avoiding misrouted corticofugal projections from the cortical germinal pathway (Bagnard et al., 1998; Lett et al., 2009).

### Pathway finding in PSPB

Pallial-subpallial boundaries (PSPB) are also known as cortical-striatal boundaries, a region separating the neocortex from the striatum. The structure of PSPB itself is thought to be important for the guidance of CT neurons because it not only functions as a structural cue but also expresses a lot of molecular guidance cues (Molnar and Cordery, 1999; Carney et al., 2009). In PSPB, an abrupt turn is made in the corticothalamic axons from ventrolateral to medial track towards the subpallium (Molnar and Cordery, 1999). PSPB is also characterized by different genes expression gradient including high in dorsal pallium to low in subpallium of *Pax6*, as well as complimentary expression pattern of *Gsh2* (Carney et al., 2009). Previous study shows that *Pax6* functions during the formation of CT connections in PSPB. In *Pax6* ^−/−^ null mutants, the outgrowth of cortical axons is not affected at E14.5, but fail to invade the internal capsule through the PSPB due to the abnormally densely-packed cells. Up to E16.5, the axons begin to misroute towards the lateral striatum but avoid entering the medal capsule and the corridor. By E18.5, most of the CT axons follow an ectopic trace along the basal surface, but some caudally-located axons go through the PSPB and follow a more lateral pathway, as a result, they do not reach the thalamus (Pratt et al., 2000; Jones et al., 2002).

### Guidance of CT axons in the subpallium

After going through the PSPB, the CT axons enter the subpallium. One surprising behavior of CT axons is a one-day pause in the subpallium of the lateral striatum (McConnell et al., 1989; Jacobs et al., 2007; Deck et al., 2013). Detailed study demonstrates that this “first waiting period” during E13.5 to E14.5 involves Sema3E/PlexinD1 signaling, without which (mutations of *Sema3E* ^−/−^ or *PlexinD1* ^−/−^) the CT axons would be misled to the globus pallidus (GP) or the cerebral peduncle (CP), a corticosubcerebral axons (CSA)-like trajectory originating from layer V. Besides the spatial defect, the mutations are temporally disordered with axons arriving at the target as early as E14.5 in immaturity. These evidences suggest that the Sema3E/PlexinD1 signaling pathway plays an important role in the CT axon path-finding and the “first waiting period” by inhibition or repulsion (Chauvet et al., 2007; Deck et al., 2013). Another study on *Robo1* knock-out mouse discovers that CT axons arrive at their destination at least one day earlier prenatally, implying the temporal role of this gene independent of the robo/slit signaling pathway (See below) (Andrews et al., 2006). After the “first waiting period”, CT axons continue to grow into the subpallium where TC axons also come and they encounter each other. The current hypothesis is that the “handshake” of pioneer CT axons and TC axons takes place in the subpallium and guides each other into their proper pathways (Hevner et al., 2002) (See details in **“Handshake” modal cues** session). Besides the “first waiting period” related guidance cues and the putative “handshake” cues, in *Brn4* ^*Cre*^;*Wnt3a* ^*DTA*^ mutant mice model, in which TC projections are completely ablated because no specific markers expressing on the pathway are detected, also displays an undoubted misleading phenotype that all the CT neurons go through GP and finally reach CP (Deck et al., 2013).

### Grow into internal capsule and corridor

Netrin-1, expresses in the internal capsule (IC) and ventral telencephalon (vTel), attracts DDC positive CT axons and guides them to grow into the IC (Oeschger et al., 2012). Here, CT axons and CAS axons separate into distinguish bundles and make the way to their intended target nuclei. Shortly after entering the internal capsule, CT axons enter the corridor in the internal capsule and CAS axons pass on to the GP and finally enter the CP (Deck et al., 2013). Again, CT axons made another re-routing towards vTel, which serves as the basis of relay nuclei targeting at the prethalamus (Metin et al., 1997). After that, the CT axons confront with diencephalon–telencephalon boundary (DTB). Pathfinding during this course is complicated with a lot of molecular guidance cues involved including *FZ3*, *Celsr3*, *Ebf1*, *Dlx1*, *Dlx2*, *Pax6*, *Emx2* and *baffled*, all of which are described in detail below. Notably, these molecular guidance cues are functionally independent of each other, demonstrating that several pathways take part in the process of CT pathfinding in the internal capsule and corridor.

Planar cell polarity genes (PCP) including *frizzled3* (*FZ3*) and *Celsr3* are verified to function during CT axon pathfinding in the internal capsule. The expression of FZ3 and Celsr3 mostly overlap in mouse CNS (Tissir and Goffinet, 2006). Studies demonstrate that *FZ3* ^−/−^ and *Celsr3* ^−/−^ null mice have a similar phenotype that has a defect in axon tract including CT and TC pathway finding (Wang et al., 2002b; Tissir et al., 2005). CT axons disappear in *FZ3* forebrain neuron conditional knock out mice but no obvious defect emerges on CT axons pathfinding in the cortical or thalamic neurons in *FZ3* cKO mice model, implying that the vanished CT axons are not due to the loss of originating neurons (Hua et al., 2014). Further study illustrates the developmental defects in corridor cells in *FZ3* forebrain neuron cKO mice (Morello et al., 2015). Whether the failure of CT neuronal routing is a direct result of defective corridor cells is still unclear. Similarly, in *Celsr3* ^−/−^ knockout mouse, CT neurons fail to enter the ganglionic eminence and thus cannot enter the prethalamus either (Tissir et al., 2005).

*Ebf1* encodes an HLH transcription factor (Wang and Reed, 1993; Dubois and Vincent, 2001). In *Ebf1* null mutants, the differentiation of cells in IC is irregular during the embryonic stage (Garel et al., 1999). Meanwhile, CT axons shift to a more medial position in dorsal thalamus in the embryonic stage, which may due to an improper turn within the internal capsule. In spite of the absence of *Ebf1* gene, no change is detected on the structure of neocortex, dorsal thalamus or even expression profiles of molecular guidance cues such as *Cdh6*, *Coup-tfI*. But *Ebf1* knockout mice have a great reduction in the expression of Sema6a in a subset of cells located in the basal ganglia (Garel et al., 2002). Hence, it is likely that disturbed structures of the basal ganglia could result in a similar phenotype as that in *Ebf1* null mutants. Indeed, homeodomain transcription factors *Dlx1* and *Dlx2* knockout mice show abnormal differentiation of basal ganglia cells, and CT axons approach to the pial surface instead of a sudden turn to the basal ganglia as in control mice (Bulfone et al., 1993; Anderson et al., 1997; Qiu et al., 1997a; Garel et al., 2002). In these mutants, the defect in basal ganglia probably gives rise to the misrouted CT axons.

Studies in *Emx2* ^−/−^ null mutants demonstrate that the projecting neurons originating from layer VI are ectopically guided along the boundary of the cortex and striatum with a wider distribution compared to the axon bundle observed in control mice. Because of the outer drift trajectory, axons reach the ventral edge of telencephalon-diencephalon. As described in the TC pathway finding part of this review, the possible mechanism for CT abnormality is also due to the fact that cells in the internal capsule are displaced (Bishop et al., 2000; Lopez-Bendito et al., 2002).

In baffled mutant mice, delayed, disorganized and overfasciculated CT neurons are observed in the IC. Growth experiments *in vitro* demonstrate *baffled* mutants are slower in axon growth and shorter in axon length, which is due to a delay of more than one day in CT growth (Favero et al., 2013).

The restriction of CT axons in the IC and the induction of their re-orientation towards the vTel are partially due to the repulsion arising from Sema5B (Lett et al., 2009; Leyva-Diaz and Lopez-Bendito, 2013). It is reported that knocking down or ectopic expression of Sema5B both change the trajectory of CT axons along the new border where Sema5B expresses (Lett et al., 2009).

### Targeting at the final destination by CT axons

Once reaching the DTB, CT axons enter the prethalamus and interact with RTN cells at around E16. During this last stage in pathfinding for CT axons, Slit/Robo signaling plays an important role. Slit1 and Slit2 are expressed at the medial edge of the midbrain in overlap domains which prevents Robo positive CT axons from crossing the midline to another hemisphere through repulsion (Long et al., 2004). In *Robo1* ^−/−^;*Robo2* ^−/−^ double knock out mutants, CT axons separate into two groups, one group targeting at the prethalamus but in a more ventral path; the other group which contains the majority of CT neurons crossing the midline after going through the internal capsule nuclei. Strikingly, some of the abnormally crossed CT axons turn back to cross the midline again with unknown reasons (Lopez-Bendito et al., 2007). In *Slit2* ^−/−^ single mutant and *Slit1* ^−/−^;*Slit2* ^−/−^ double mutants, similar phenotypes are observed as in *Robo* null mutants where CT axons cross the midline into the contralateral brain (Bagri et al., 2002). Before innervating the dLGN, CT axons experience the “second waiting period” for a while. However, the spatiotemporal regulation mechanism remains largely unclear and the only known molecule to repel the innervation of CT neurons is Aggrecan (Brooks et al., 2013). The expression concentration of this protein is found negatively correlated to the innervations of CT axons. Digestion of this protein advances CT axon innervations temporally, and further studies found that retinal waves could regulate the degradation of Aggrecan (Brooks et al., 2013).

### “Handshake” cues

**Figure 7:**
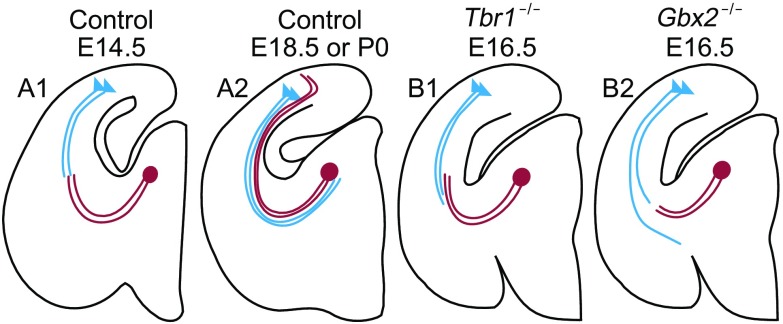
**Schematics for handshake model and related guidance cues**. (A1–2) Schematics for handshake model. (B1–2) Misled CT and TC axons after *Tbr1* and *Gbx2* genes knock-out. Blue triangles represent corticothalamic projecting neurons. Blue lines represent CT axons. Red dots represent thalamocortical projecting neurons. Red lines represent TC axons. CP: cerebral peduncle

Several mouse lines provide evidences for the proposal of “handshake” model in the internal capsule, where pioneer TC and CT axons encounter and interact to guide each other. After that, pioneer TC and CT axons continue to grow in opposite directions and reach their targets. Here, studies on some transgenic mice lines like *Tbr1* and *Gbx2* shed light on the “handshake” assumption (Hevner et al., 2002) (Fig. 7).

*Tbr1*, T-box transcription factor gene, expresses in the cortex including layer VI and subplate neurons but not in the thalamus. In *Tbr1* null mutants, thalamic axons deviate into the external capsule region without entering the cortex. Meanwhile, corticothalamic axons terminate in the internal capsule and never grow into the dorsal thalamus (Hevner et al., 2002). The evidence of *Tbr1*’s function of fate determination in layer VI (See detailed description in **Fate determine and outgrowth of neocortex** session) may suggest the possibility that *Tbr1* determines the projection fate of layer VI neurons and in *Tbr1* knockout mice, the CT axons grow out of their intended trajectories. On losing the “handshake” guidance in the internal capsule, TC axons also fail to reach the proper cortical area (Hevner et al., 2002; McKenna et al., 2011).

The *Gbx2* homeobox gene is expressed in the dorsal thalamic neurons during the early development stage, but not in the cortex (Jones and Rubenstein, 2004; Chen et al., 2009). In most *Gbx2* ^−/−^ mutants, thalamocortical axons decrease in number and grow no farther than the internal capsule (subpallium). As for the corticothalamic axons, all of them originate from neurons of layer VI in the cortex and arrive at the subpallial telencephalon as in control mice at E14.5. However, axons are observed to grow into the cerebral peduncle but unable to be detected in the dorsal thalamus at E16.5, a time point when some pioneer CT axons have reached the dorsal thalamus. This misrouted guidance may due to the loss of guidance by TC axons, or the loss of molecular identity in the thalamus after *Gbx2* deletion alternatively. In most cases, no axons invade into the thalamus by E18.5 except for one mouse in which very sparse CT and TC axons are detected simultaneously (Mallika et al., 2015). Two recent studies show a dramatic loss of the cortex from E16.5 and a total loss of CT projections in *Tra2β* conditional knockout mice, exhibiting not only mistargeted TC projections but also missing of the retinogeniculate projections(Shanks et al., 2016; Diao et al., 2017). One possible explanation for the misled TC in *Tra2β* knock out mice is the disrupted “handshake” process due to the loss of V1.

## Conclusion and future direction

Molecular guidance cues play a constructive role on guiding several streams of axons to target at the right position along the visual pathway. Different molecular cues function through diverse mechanisms including concentration gradient, area specific or cell type specific expression, repellent or attractive ligand and receptors, and molecules regulated by neuronal activities. Concentration gradient is the most popular model in the development and patterning of organisms, as in neuronal development, unbalanced expression of cues within a certain territory usually provide growth guidance. Throughout the retina-dLGN-V1-dLGN circuit, many molecules guide the growth of axons via mechanisms such as CS-PG, *Foxd1*, Nr-CAM, *Ten-m3*, *p75NTR*, *ephrinA2*/*A3*/*A5*, *Pax6*. Similar to the gradient, a lot of area- or cell type-specific guidance cues participate in guiding visual axons including *Zic2*, *Brn3b*, *Brn3c*, *CD44*, *Isl2*, *Lhx2*, *Ebf1*, *Dlx1*/*2*, *Ngn2* and *Tbr1*. Ligands such as EphB1/epherin-B2, Sema3E/PlexinD1 and Slit/Robo that are repellent or attractive to their corresponding receptors also play roles in the axon guidance. Besides, NPs, MHCI, and Aggrecan emerge along the retina-dLGN-V1 pathway, where they interact with neuronal activity and participate in the visual development and wiring.

In the past decades, a lot of studies focused on these guidance cues singly or doubly using genetically knock-out mice or blocking technology, and little attention is paid to the relationship between these guidance cues or the pathways related to these cues. Now, high throughput methods may provide opportunities to figure out the whole network of the guidance cues for a better understanding of how the guidance cues instruct axons to be wired precisely step by step. The interaction between the neuronal activity and the molecules is also involved in axon guidance. In this review, a handful of molecules such as MHCI family (Corriveau et al., 1998), NPs (Tsui et al., 1996) and Aggrecan (Brooks et al., 2013) are modulated by neuronal activity or retina waves. MHCI family and NPs link to function in synaptic wiring or plasticity. However, the Aggrecan protein is down-regulated by retinal waves which perturb the innervation time of corticothalamic axons. Concluded from these studies, the diverse and dynamic interactions between neuronal activity and molecules jointly make excellent networks for input and output of visual information. New molecular guidance cues remain to be discovered, aiming at a more comprehensive understanding of the mechanisms of pathfinding on the visual pathway.

## References

[CR1] Anderson SA, Qiu M, Bulfone A, Eisenstat DD, Meneses J, Pedersen R, Rubenstein JL (1997). Mutations of the homeobox genes Dlx-1 and Dlx-2 disrupt the striatal subventricular zone and differentiation of late born striatal neurons. Neuron.

[CR2] Andrews W, Liapi A, Plachez C, Camurri L, Zhang J, Mori S, Murakami F, Parnavelas JG, Sundaresan V, Richards LJ (2006). Robo1 regulates the development of major axon tracts and interneuron migration in the forebrain. Development.

[CR3] Bagnard D, Lohrum M, Uziel D, Puschel AW, Bolz J (1998). Semaphorins act as attractive and repulsive guidance signals during the development of cortical projections. Development.

[CR4] Bagri A, Marin O, Plump AS, Mak J, Pleasure SJ, Rubenstein JL, Tessier-Lavigne M (2002). Slit proteins prevent midline crossing and determine the dorsoventral position of major axonal pathways in the mammalian forebrain. Neuron.

[CR5] Bandtlow CE, Zimmermann DR (2000). Proteoglycans in the developing brain: new conceptual insights for old proteins. Physiol Rev.

[CR6] Bertuzzi S, Hindges R, Mui SH, O’Leary DD, Lemke G (1999). The homeodomain protein vax1 is required for axon guidance and major tract formation in the developing forebrain. Genes Dev.

[CR7] Bishop KM, Goudreau G, O’Leary DD (2000). Regulation of area identity in the mammalian neocortex by Emx2 and Pax6. Science.

[CR8] Bjartmar L, Huberman AD, Ullian EM, Renteria RC, Liu X, Xu W, Prezioso J, Susman MW, Stellwagen D, Stokes CC (2006). Neuronal pentraxins mediate synaptic refinement in the developing visual system. J Neurosci.

[CR9] Bloom AJ, Miller BR, Sanes JR, DiAntonio A (2007). The requirement for Phr1 in CNS axon tract formation reveals the corticostriatal boundary as a choice point for cortical axons. Genes Dev.

[CR10] Borrell V, Del Rio JA, Alcantara S, Derer M, Martinez A, D’Arcangelo G, Nakajima K, Mikoshiba K, Derer P, Curran T (1999). Reelin regulates the development and synaptogenesis of the layer-specific entorhino-hippocampal connections. J Neurosci.

[CR11] Brooks JM, Su J, Levy C, Wang JS, Seabrook TA, Guido W, Fox MA (2013). A molecular mechanism regulating the timing of corticogeniculate innervation. Cell Rep.

[CR12] Bulfone A, Puelles L, Porteus MH, Frohman MA, Martin GR, Rubenstein JL (1993). Spatially restricted expression of Dlx-1, Dlx-2 (Tes-1), Gbx-2, and Wnt-3 in the embryonic day 12.5 mouse forebrain defines potential transverse and longitudinal segmental boundaries. J Neurosci.

[CR13] Bulfone A, Martinez S, Marigo V, Campanella M, Basile A, Quaderi N, Gattuso C, Rubenstein JL, Ballabio A (1999). Expression pattern of the Tbr2 (Eomesodermin) gene during mouse and chick brain development. Mech Dev.

[CR14] Burgess RW, Peterson KA, Johnson MJ, Roix JJ, Welsh IC, O’Brien TP (2004). Evidence for a conserved function in synapse formation reveals Phr1 as a candidate gene for respiratory failure in newborn mice. Mol Cell Biol.

[CR15] Cang J, Feldheim DA (2013). Developmental mechanisms of topographic map formation and alignment. Annu Rev Neurosci.

[CR16] Carney RS, Cocas LA, Hirata T, Mansfield K, Corbin JG (2009). Differential regulation of telencephalic pallial-subpallial boundary patterning by Pax6 and Gsh2. Cereb Cortex.

[CR17] Carr OP, Glendining KA, Leamey CA, Marotte LR (2014). Retinal overexpression of Ten-m3 alters ipsilateral retinogeniculate projections in the wallaby (*Macropus eugenii*). Neurosci Lett.

[CR18] Carreres MI, Escalante A, Murillo B, Chauvin G, Gaspar P, Vegar C, Herrera E (2011). Transcription factor Foxd1 is required for the specification of the temporal retina in mammals. J Neurosci.

[CR19] Cecchi C (2002). Emx2: a gene responsible for cortical development, regionalization and area specification. Gene.

[CR20] Chao HT, Zoghbi HY, Rosenmund C (2007). MeCP2 controls excitatory synaptic strength by regulating glutamatergic synapse number. Neuron.

[CR21] Chapman B, Stryker MP (1993). Development of orientation selectivity in ferret visual cortex and effects of deprivation. J Neurosci.

[CR22] Chauvet S, Cohen S, Yoshida Y, Fekrane L, Livet J, Gayet O, Segu L, Buhot MC, Jessell TM, Henderson CE (2007). Gating of Sema3E/PlexinD1 signaling by neuropilin-1 switches axonal repulsion to attraction during brain development. Neuron.

[CR23] Chen L, Guo Q, Li JY (2009). Transcription factor Gbx2 acts cell-nonautonomously to regulate the formation of lineage-restriction boundaries of the thalamus. Development.

[CR24] Chew KS, Renna JM, McNeill DS, Fernandez DC, Keenan WT, Thomsen MB, Ecker JL, Loevinsohn GS, VanDunk C, Vicarel DC (2017). A subset of ipRGCs regulates both maturation of the circadian clock and segregation of retinogeniculate projections in mice. Elife.

[CR25] Chung KY, Taylor JS, Shum DK, Chan SO (2000). Axon routing at the optic chiasm after enzymatic removal of chondroitin sulfate in mouse embryos. Development.

[CR26] Colello RJ, Guillery RW (1990). The early development of retinal ganglion cells with uncrossed axons in the mouse: retinal position and axonal course. Development.

[CR27] Corriveau RA, Huh GS, Shatz CJ (1998). Regulation of class I MHC gene expression in the developing and mature CNS by neural activity. Neuron.

[CR28] Culican SM, Bloom AJ, Weiner JA, DiAntonio A (2009). Phr1 regulates retinogeniculate targeting independent of activity and ephrin-A signalling. Mol Cell Neurosci.

[CR29] D’Arcangelo G, Miao GG, Chen SC, Soares HD, Morgan JI, Curran T (1995). A protein related to extracellular matrix proteins deleted in the mouse mutant reeler. Nature.

[CR30] D’Arcangelo G, Nakajima K, Miyata T, Ogawa M, Mikoshiba K, Curran T (1997). Reelin is a secreted glycoprotein recognized by the CR-50 monoclonal antibody. J Neurosci.

[CR31] Datwani A, McConnell MJ, Kanold PO, Micheva KD, Busse B, Shamloo M, Smith SJ, Shatz CJ (2009). Classical MHCI molecules regulate retinogeniculate refinement and limit ocular dominance plasticity. Neuron.

[CR32] Deck M, Lokmane L, Chauvet S, Mailhes C, Keita M, Niquille M, Yoshida M, Yoshida Y, Lebrand C, Mann F (2013). Pathfinding of corticothalamic axons relies on a rendezvous with thalamic projections. Neuron.

[CR33] Dhande OS, Bhatt S, Anishchenko A, Elstrott J, Iwasato T, Swindell EC, Xu HP, Jamrich M, Itohara S, Feller MB (2012). Role of adenylate cyclase 1 in retinofugal map development. J Comp Neurol.

[CR34] DiAntonio A, Haghighi AP, Portman SL, Lee JD, Amaranto AM, Goodman CS (2001). Ubiquitination-dependent mechanisms regulate synaptic growth and function. Nature.

[CR35] Diao Y, Cui L, Chen Y, Burbridge TJ, Han W, Wirth B, Sestan N, Crair MC, Zhang J (2017). Reciprocal connections between cortex and thalamus contribute to retinal axon targeting to dorsal lateral geniculate nucleus. Cereb Cortex.

[CR36] Dodds DC, Omeis IA, Cushman SJ, Helms JA, Perin MS (1997). Neuronal pentraxin receptor, a novel putative integral membrane pentraxin that interacts with neuronal pentraxin 1 and 2 and taipoxin-associated calcium-binding protein 49. J Biol Chem.

[CR37] Drager UC (1985). Birth dates of retinal ganglion cells giving rise to the crossed and uncrossed optic projections in the mouse. Proc R Soc Lond B Biol Sci.

[CR38] Drager UC, Olsen JF (1980). Origins of crossed and uncrossed retinal projections in pigmented and albino mice. J Comp Neurol.

[CR39] D’Souza J, Hendricks M, Le Guyader S, Subburaju S, Grunewald B, Scholich K, Jesuthasan S (2005). Formation of the retinotectal projection requires Esrom, an ortholog of PAM (protein associated with Myc). Development.

[CR40] Dubois L, Vincent A (2001). The COE–Collier/Olf1/EBF–transcription factors: structural conservation and diversity of developmental functions. Mech Dev.

[CR41] Dufour A, Seibt J, Passante L, Depaepe V, Ciossek T, Frisen J, Kullander K, Flanagan JG, Polleux F, Vanderhaeghen P (2003). Area specificity and topography of thalamocortical projections are controlled by ephrin/Eph genes. Neuron.

[CR42] Egea J, Klein R (2007). Bidirectional Eph-ephrin signaling during axon guidance. Trends Cell Biol.

[CR43] Erkman L, Yates PA, McLaughlin T, McEvilly RJ, Whisenhunt T, O’Connell SM, Krones AI, Kirby MA, Rapaport DH, Bermingham JR (2000). A POU domain transcription factor-dependent program regulates axon pathfinding in the vertebrate visual system. Neuron.

[CR44] Erskine L, Williams SE, Brose K, Kidd T, Rachel RA, Goodman CS, Tessier-Lavigne M, Mason CA (2000). Retinal ganglion cell axon guidance in the mouse optic chiasm: expression and function of robos and slits. J Neurosci.

[CR45] Favero CB, Henshaw RN, Grimsley-Myers CM, Shrestha A, Beier DR, Dwyer ND (2013). Mutation of the BiP/GRP78 gene causes axon outgrowth and fasciculation defects in the thalamocortical connections of the mammalian forebrain. J Comp Neurol.

[CR46] Fonseca M, del Rio JA, Martinez A, Gomez S, Soriano E (1995). Development of calretinin immunoreactivity in the neocortex of the rat. J Comp Neurol.

[CR47] Formstone CJ, Little PF (2001). The flamingo-related mouse Celsr family (Celsr1-3) genes exhibit distinct patterns of expression during embryonic development. Mech Dev.

[CR48] Fukuda T, Itoh M, Ichikawa T, Washiyama K, Goto Y (2005). Delayed maturation of neuronal architecture and synaptogenesis in cerebral cortex of Mecp2-deficient mice. J Neuropathol Exp Neurol.

[CR49] Gan L, Wang SW, Huang Z, Klein WH (1999). POU domain factor Brn-3b is essential for retinal ganglion cell differentiation and survival but not for initial cell fate specification. Dev Biol.

[CR50] Garel S, Marin F, Grosschedl R, Charnay P (1999). Ebf1 controls early cell differentiation in the embryonic striatum. Development.

[CR51] Garel S, Yun K, Grosschedl R, Rubenstein JL (2002). The early topography of thalamocortical projections is shifted in Ebf1 and Dlx1/2 mutant mice. Development.

[CR52] Gebhardt C, Bastmeyer M, Weth F (2012). Balancing of ephrin/Eph forward and reverse signaling as the driving force of adaptive topographic mapping. Development.

[CR53] Gonthier B, Nasarre C, Roth L, Perraut M, Thomasset N, Roussel G, Aunis D, Bagnard D (2007). Functional interaction between matrix metalloproteinase-3 and semaphorin-3C during cortical axonal growth and guidance. Cereb Cortex.

[CR54] Grinberg I, Millen KJ (2005). The ZIC gene family in development and disease. Clin Genet.

[CR55] Guillery RW, Mason CA, Taylor JS (1995). Developmental determinants at the mammalian optic chiasm. J Neurosci.

[CR56] Gulisano M, Broccoli V, Pardini C, Boncinelli E (1996). Emx1 and Emx2 show different patterns of expression during proliferation and differentiation of the developing cerebral cortex in the mouse. Eur J Neurosci.

[CR57] Hagman J, Belanger C, Travis A, Turck CW, Grosschedl R (1993). Cloning and functional characterization of early B-cell factor, a regulator of lymphocyte-specific gene expression. Genes Dev.

[CR58] Herrera E, Brown L, Aruga J, Rachel RA, Dolen G, Mikoshiba K, Brown S, Mason CA (2003). Zic2 patterns binocular vision by specifying the uncrossed retinal projection. Cell.

[CR59] Herrera E, Marcus R, Li S, Williams SE, Erskine L, Lai E, Mason C (2004). Foxd1 is required for proper formation of the optic chiasm. Development.

[CR60] Hevner RF, Shi L, Justice N, Hsueh Y, Sheng M, Smiga S, Bulfone A, Goffinet AM, Campagnoni AT, Rubenstein JL (2001). Tbr1 regulates differentiation of the preplate and layer 6. Neuron.

[CR61] Hevner RF, Miyashita-Lin E, Rubenstein JL (2002). Cortical and thalamic axon pathfinding defects in Tbr1, Gbx2, and Pax6 mutant mice: evidence that cortical and thalamic axons interact and guide each other. J Comp Neurol.

[CR62] Howell BW, Hawkes R, Soriano P, Cooper JA (1997). Neuronal position in the developing brain is regulated by mouse disabled-1. Nature.

[CR63] Hua ZL, Jeon S, Caterina MJ, Nathans J (2014). Frizzled3 is required for the development of multiple axon tracts in the mouse central nervous system. Proc Natl Acad Sci USA.

[CR64] Huberman AD, Speer CM, Chapman B (2006). Spontaneous retinal activity mediates development of ocular dominance columns and binocular receptive fields in v1. Neuron.

[CR65] Huberman AD, Feller MB, Chapman B (2008). Mechanisms underlying development of visual maps and receptive fields. Annu Rev Neurosci.

[CR67] Huh GS, Boulanger LM, Du H, Riquelme PA, Brotz TM, Shatz CJ (2000). Functional requirement for class I MHC in CNS development and plasticity. Science.

[CR68] International Schizophrenia Consortium, Purcell SM, Wray NR, Stone JL., Visscher PM, O’Donovan MC, Sullivan PF, Sklar P (2009). Common polygenic variation contributes to risk of schizophrenia and bipolar disorder. Nature.

[CR69] Jacobs EC, Campagnoni C, Kampf K, Reyes SD, Kalra V, Handley V, Xie YY, Hong-Hu Y, Spreur V, Fisher RS (2007). Visualization of corticofugal projections during early cortical development in a tau-GFP-transgenic mouse. Eur J Neurosci.

[CR70] Jones EG, Rubenstein JL (2004). Expression of regulatory genes during differentiation of thalamic nuclei in mouse and monkey. J Comp Neurol.

[CR71] Jones L, Lopez-Bendito G, Gruss P, Stoykova A, Molnar Z (2002). Pax6 is required for the normal development of the forebrain axonal connections. Development.

[CR72] Kim N, Min KW, Kang KH, Lee EJ, Kim HT, Moon K, Choi J, Le D, Lee SH, Kim JW (2014). Regulation of retinal axon growth by secreted Vax1 homeodomain protein. Elife.

[CR73] Koch SM, Ullian EM (2010). Neuronal pentraxins mediate silent synapse conversion in the developing visual system. J Neurosci.

[CR74] Kruger K, Tam AS, Lu C, Sretavan DW (1998). Retinal ganglion cell axon progression from the optic chiasm to initiate optic tract development requires cell autonomous function of GAP-43. J Neurosci.

[CR75] Lakhina V, Falnikar A, Bhatnagar L, Tole S (2007). Early thalamocortical tract guidance and topographic sorting of thalamic projections requires LIM-homeodomain gene Lhx2. Dev Biol.

[CR76] Leamey CA, Merlin S, Lattouf P, Sawatari A, Zhou X, Demel N, Glendining KA, Oohashi T, Sur M, Fassler R (2007). Ten_m3 regulates eye-specific patterning in the mammalian visual pathway and is required for binocular vision. PLoS Biol.

[CR77] Leamey CA, Glendining KA, Kreiman G, Kang ND, Wang KH, Fassler R, Sawatari A, Tonegawa S, Sur M (2008). Differential gene expression between sensory neocortical areas: potential roles for Ten_m3 and Bcl6 in patterning visual and somatosensory pathways. Cereb Cortex.

[CR78] Lett RL, Wang W, O’Connor TP (2009). Semaphorin 5B is a novel inhibitory cue for corticofugal axons. Cereb Cortex.

[CR79] Leung KM, Margolis RU, Chan SO (2004). Expression of phosphacan and neurocan during early development of mouse retinofugal pathway. Brain Res Dev Brain Res.

[CR80] Lewcock JW, Genoud N, Lettieri K, Pfaff SL (2007). The ubiquitin ligase Phr1 regulates axon outgrowth through modulation of microtubule dynamics. Neuron.

[CR81] Lewis JD, Meehan RR, Henzel WJ, Maurer-Fogy I, Jeppesen P, Klein F, Bird A (1992). Purification, sequence, and cellular localization of a novel chromosomal protein that binds to methylated DNA. Cell.

[CR82] Leyva-Diaz E, Lopez-Bendito G (2013). In and out from the cortex: development of major forebrain connections. Neuroscience.

[CR83] Lin L, Chan SO (2003). Perturbation of CD44 function affects chiasmatic routing of retinal axons in brain slice preparations of the mouse retinofugal pathway. Eur J Neurosci.

[CR84] Little GE, Lopez-Bendito G, Runker AE, Garcia N, Pinon MC, Chedotal A, Molnar Z, Mitchell KJ (2009). Specificity and plasticity of thalamocortical connections in Sema6A mutant mice. PLoS Biol.

[CR85] Long H, Sabatier C, Ma L, Plump A, Yuan W, Ornitz DM, Tamada A, Murakami F, Goodman CS, Tessier-Lavigne M (2004). Conserved roles for Slit and Robo proteins in midline commissural axon guidance. Neuron.

[CR86] Lopez-Bendito G, Molnar Z (2003). Thalamocortical development: how are we going to get there?. Nat Rev Neurosci.

[CR87] Lopez-Bendito G, Chan CH, Mallamaci A, Parnavelas J, Molnar Z (2002). Role of Emx2 in the development of the reciprocal connectivity between cortex and thalamus. J Comp Neurol.

[CR88] Lopez-Bendito G, Flames N, Ma L, Fouquet C, Di Meglio T, Chedotal A, Tessier-Lavigne M, Marin O (2007). Robo1 and Robo2 cooperate to control the guidance of major axonal tracts in the mammalian forebrain. J Neurosci.

[CR89] Lustig M, Sakurai T, Grumet M (1999). Nr-CAM promotes neurite outgrowth from peripheral ganglia by a mechanism involving axonin-1 as a neuronal receptor. Dev Biol.

[CR90] Lustig M, Erskine L, Mason CA, Grumet M, Sakurai T (2001). Nr-CAM expression in the developing mouse nervous system: ventral midline structures, specific fiber tracts, and neuropilar regions. J Comp Neurol.

[CR91] Magnani D, Morle L, Hasenpusch-Theil K, Paschaki M, Jacoby M, Schurmans S, Durand B, Theil T (2015). The ciliogenic transcription factor Rfx3 is required for the formation of the thalamocortical tract by regulating the patterning of prethalamus and ventral telencephalon. Hum Mol Genet.

[CR92] Mallamaci A, Muzio L, Chan CH, Parnavelas J, Boncinelli E (2000). Area identity shifts in the early cerebral cortex of Emx2-/- mutant mice. Nat Neurosci.

[CR93] Mallika C, Guo Q, Li JY (2015). Gbx2 is essential for maintaining thalamic neuron identity and repressing habenular characters in the developing thalamus. Dev Biol.

[CR94] Marcus RC, Shimamura K, Sretavan D, Lai E, Rubenstein JL, Mason CA (1999). Domains of regulatory gene expression and the developing optic chiasm: correspondence with retinal axon paths and candidate signaling cells. J Comp Neurol.

[CR95] Matsuki T, Matthews RT, Cooper JA, van der Brug MP, Cookson MR, Hardy JA, Olson EC, Howell BW (2010). Reelin and stk25 have opposing roles in neuronal polarization and dendritic Golgi deployment. Cell.

[CR96] McConnell SK, Ghosh A, Shatz CJ (1989). Subplate neurons pioneer the first axon pathway from the cerebral cortex. Science.

[CR97] McKenna WL, Betancourt J, Larkin KA, Abrams B, Guo C, Rubenstein JL, Chen B (2011). Tbr1 and Fezf2 regulate alternate corticofugal neuronal identities during neocortical development. J Neurosci.

[CR98] McLaughlin T, O’Leary DD (2005). Molecular gradients and development of retinotopic maps. Annu Rev Neurosci.

[CR99] McQuillen PS, DeFreitas MF, Zada G, Shatz CJ (2002). A novel role for p75NTR in subplate growth cone complexity and visual thalamocortical innervation. J Neurosci.

[CR100] Metin C, Deleglise D, Serafini T, Kennedy TE, Tessier-Lavigne M (1997). A role for netrin-1 in the guidance of cortical efferents. Development.

[CR101] Molnar Z, Cordery P (1999). Connections between cells of the internal capsule, thalamus, and cerebral cortex in embryonic rat. J Comp Neurol.

[CR102] Morello F, Prasad AA, Rehberg K, Vieira de Sa R, Anton-Bolanos N, Leyva-Diaz E, Adolfs Y, Tissir F, Lopez-Bendito G, Pasterkamp RJ (2015). Frizzled3 controls axonal polarity and intermediate target entry during striatal pathway development. J Neurosci.

[CR103] Morin LP, Studholme KM (2014). Retinofugal projections in the mouse. J Comp Neurol.

[CR104] Muir-Robinson G, Hwang BJ, Feller MB (2002). Retinogeniculate axons undergo eye-specific segregation in the absence of eye-specific layers. J Neurosci.

[CR105] Nagai T, Aruga J, Minowa O, Sugimoto T, Ohno Y, Noda T, Mikoshiba K (2000). Zic2 regulates the kinetics of neurulation. Proc Natl Acad Sci USA.

[CR106] Nakagawa Y, O’Leary DD (2001). Combinatorial expression patterns of LIM-homeodomain and other regulatory genes parcellate developing thalamus. J Neurosci.

[CR107] Nakagawa S, Brennan C, Johnson KG, Shewan D, Harris WA, Holt CE (2000). Ephrin-B regulates the Ipsilateral routing of retinal axons at the optic chiasm. Neuron.

[CR108] Niclou SP, Jia L, Raper JA (2000). Slit2 is a repellent for retinal ganglion cell axons. J Neurosci.

[CR109] Nicol X, Bennis M, Ishikawa Y, Chan GC, Reperant J, Storm DR, Gaspar P (2006). Role of the calcium modulated cyclases in the development of the retinal projections. Eur J Neurosci.

[CR110] Nicol X, Muzerelle A, Rio JP, Metin C, Gaspar P (2006). Requirement of adenylate cyclase 1 for the ephrin-A5-dependent retraction of exuberant retinal axons. J Neurosci.

[CR111] Noutel J, Hong YK, Leu B, Kang E, Chen C (2011). Experience-dependent retinogeniculate synapse remodeling is abnormal in MeCP2-deficient mice. Neuron.

[CR112] Oeschger FM, Wang WZ, Lee S, Garcia-Moreno F, Goffinet AM, Arbones ML, Rakic S, Molnar Z (2012). Gene expression analysis of the embryonic subplate. Cereb Cortex.

[CR113] O’Leary DM, Gerfen CR, Cowan WM (1983). The development and restriction of the ipsilateral retinofugal projection in the chick. Brain Res.

[CR114] Oohashi T, Zhou XH, Feng K, Richter B, Morgelin M, Perez MT, Su WD, Chiquet-Ehrismann R, Rauch U, Fassler R (1999). Mouse ten-m/Odz is a new family of dimeric type II transmembrane proteins expressed in many tissues. J Cell Biol.

[CR115] Pak W, Hindges R, Lim YS, Pfaff SL, O’Leary DD (2004). Magnitude of binocular vision controlled by islet-2 repression of a genetic program that specifies laterality of retinal axon pathfinding. Cell.

[CR116] Panza P, Sitko AA, Maischein HM, Koch I, Flotenmeyer M, Wright GJ, Mandai K, Mason CA, Sollner C (2015). The LRR receptor Islr2 is required for retinal axon routing at the vertebrate optic chiasm. Neural Dev.

[CR117] Petros TJ, Shrestha BR, Mason C (2009). Specificity and sufficiency of EphB1 in driving the ipsilateral retinal projection. J Neurosci.

[CR118] Pfeiffenberger C, Cutforth T, Woods G, Yamada J, Renteria RC, Copenhagen DR, Flanagan JG, Feldheim DA (2005). Ephrin-As and neural activity are required for eye-specific patterning during retinogeniculate mapping. Nat Neurosci.

[CR119] Plas DT, Visel A, Gonzalez E, She WC, Crair MC (2004). Adenylate Cyclase 1 dependent refinement of retinotopic maps in the mouse. Vision Res.

[CR120] Plump AS, Erskine L, Sabatier C, Brose K, Epstein CJ, Goodman CS, Mason CA, Tessier-Lavigne M (2002). Slit1 and Slit2 cooperate to prevent premature midline crossing of retinal axons in the mouse visual system. Neuron.

[CR121] Popp S, Maurel P, Andersen JS, Margolis RU (2004). Developmental changes of aggrecan, versican and neurocan in the retina and optic nerve. Exp Eye Res.

[CR122] Pratt T, Vitalis T, Warren N, Edgar JM, Mason JO, Price DJ (2000). A role for Pax6 in the normal development of dorsal thalamus and its cortical connections. Development.

[CR123] Pratt T, Tian NM, Simpson TI, Mason JO, Price DJ (2004). The winged helix transcription factor Foxg1 facilitates retinal ganglion cell axon crossing of the ventral midline in the mouse. Development.

[CR124] Qiu M, Bulfone A, Ghattas I, Meneses JJ, Christensen L, Sharpe PT, Presley R, Pedersen RA, Rubenstein JL (1997). Role of the Dlx homeobox genes in proximodistal patterning of the branchial arches: mutations of Dlx-1, Dlx-2, and Dlx-1 and -2 alter morphogenesis of proximal skeletal and soft tissue structures derived from the first and second arches. Dev Biol.

[CR125] Qiu Y, Pereira FA, DeMayo FJ, Lydon JP, Tsai SY, Tsai MJ (1997). Null mutation of mCOUP-TFI results in defects in morphogenesis of the glossopharyngeal ganglion, axonal projection, and arborization. Genes Dev.

[CR126] Rachel RA, Dolen G, Hayes NL, Lu A, Erskine L, Nowakowski RS, Mason CA (2002). Spatiotemporal features of early neuronogenesis differ in wild-type and albino mouse retina. J Neurosci.

[CR127] Rebsam A, Petros TJ, Mason CA (2009). Switching retinogeniculate axon laterality leads to normal targeting but abnormal eye-specific segregation that is activity dependent. J Neurosci.

[CR128] Reid MS, Blobel CP (1994). Apexin, an acrosomal pentaxin. J Biol Chem.

[CR129] Rogers JT, Rusiana I, Trotter J, Zhao L, Donaldson E, Pak DT, Babus LW, Peters M, Banko JL, Chavis P (2011). Reelin supplementation enhances cognitive ability, synaptic plasticity, and dendritic spine density. Learn Mem.

[CR130] Rossi FM, Pizzorusso T, Porciatti V, Marubio LM, Maffei L, Changeux JP (2001). Requirement of the nicotinic acetylcholine receptor beta 2 subunit for the anatomical and functional development of the visual system. Proc Natl Acad Sci USA.

[CR131] Rothberg JM, Jacobs JR, Goodman CS, Artavanis-Tsakonas S (1990). slit: an extracellular protein necessary for development of midline glia and commissural axon pathways contains both EGF and LRR domains. Genes Dev.

[CR132] Royaux I, Lambert de Rouvroit C, D’Arcangelo G, Demirov D, Goffinet AM (1997). Genomic organization of the mouse reelin gene. Genomics.

[CR133] Sakurai T, Lustig M, Babiarz J, Furley AJ, Tait S, Brophy PJ, Brown SA, Brown LY, Mason CA, Grumet M (2001). Overlapping functions of the cell adhesion molecules Nr-CAM and L1 in cerebellar granule cell development. J Cell Biol.

[CR134] Schaefer AM, Hadwiger GD, Nonet ML (2000). rpm-1, a conserved neuronal gene that regulates targeting and synaptogenesis in C. elegans. Neuron.

[CR135] Schafer DP, Heller CT, Gunner G, Heller M, Gordon C, Hammond T, Wolf Y, Jung S, Stevens B (2016). Microglia contribute to circuit defects in Mecp2 null mice independent of microglia-specific loss of Mecp2 expression. Elife.

[CR136] Schlimgen AK, Helms JA, Vogel H, Perin MS (1995). Neuronal pentraxin, a secreted protein with homology to acute phase proteins of the immune system. Neuron.

[CR137] Seibt J, Schuurmans C, Gradwhol G, Dehay C, Vanderhaeghen P, Guillemot F, Polleux F (2003). Neurogenin2 specifies the connectivity of thalamic neurons by controlling axon responsiveness to intermediate target cues. Neuron.

[CR138] Shanks JA, Ito S, Schaevitz L, Yamada J, Chen B, Litke A, Feldheim DA (2016). Corticothalamic axons are essential for retinal ganglion cell axon targeting to the mouse dorsal lateral geniculate nucleus. J Neurosci.

[CR139] Shatz CJ (2009). MHC class I: an unexpected role in neuronal plasticity. Neuron.

[CR140] Sheldon M, Rice DS, D’Arcangelo G, Yoneshima H, Nakajima K, Mikoshiba K, Howell BW, Cooper JA, Goldowitz D, Curran T (1997). Scrambler and yotari disrupt the disabled gene and produce a reeler-like phenotype in mice. Nature.

[CR141] Skene JH (1990). GAP-43 as a ‘calmodulin sponge’ and some implications for calcium signalling in axon terminals. Neurosci Res Suppl.

[CR142] Snow DM, Watanabe M, Letourneau PC, Silver J (1991). A chondroitin sulfate proteoglycan may influence the direction of retinal ganglion cell outgrowth. Development.

[CR143] Sretavan DW, Kruger K (1998). Randomized retinal ganglion cell axon routing at the optic chiasm of GAP-43-deficient mice: association with midline recrossing and lack of normal ipsilateral axon turning. J Neurosci.

[CR145] Sretavan DW, Feng L, Pure E, Reichardt LF (1994). Embryonic neurons of the developing optic chiasm express L1 and CD44, cell surface molecules with opposing effects on retinal axon growth. Neuron.

[CR146] Srinivasan K, Leone DP, Bateson RK, Dobreva G, Kohwi Y, Kohwi-Shigematsu T, Grosschedl R, McConnell SK (2012). A network of genetic repression and derepression specifies projection fates in the developing neocortex. Proc Natl Acad Sci USA.

[CR147] Stevens B, Allen NJ, Vazquez LE, Howell GR, Christopherson KS, Nouri N, Micheva KD, Mehalow AK, Huberman AD, Stafford B (2007). The classical complement cascade mediates CNS synapse elimination. Cell.

[CR148] Strittmatter SM, Fankhauser C, Huang PL, Mashimo H, Fishman MC (1995). Neuronal pathfinding is abnormal in mice lacking the neuronal growth cone protein GAP-43. Cell.

[CR149] Su J, Haner CV, Imbery TE, Brooks JM, Morhardt DR, Gorse K, Guido W, Fox MA (2011). Reelin is required for class-specific retinogeniculate targeting. J Neurosci.

[CR150] Su J, Klemm MA, Josephson AM, Fox MA (2013). Contributions of VLDLR and LRP8 in the establishment of retinogeniculate projections. Neural Dev.

[CR151] Swindell EC, Bailey TJ, Loosli F, Liu C, Amaya-Manzanares F, Mahon KA, Wittbrodt J, Jamrich M (2006). Rx-Cre, a tool for inactivation of gene expression in the developing retina. Genesis.

[CR152] Syken J, Grandpre T, Kanold PO, Shatz CJ (2006). PirB restricts ocular-dominance plasticity in visual cortex. Science.

[CR153] Thaler JP, Koo SJ, Kania A, Lettieri K, Andrews S, Cox C, Jessell TM, Pfaff SL (2004). A postmitotic role for Isl-class LIM homeodomain proteins in the assignment of visceral spinal motor neuron identity. Neuron.

[CR154] Tissir F, Goffinet AM (2006). Expression of planar cell polarity genes during development of the mouse CNS. Eur J Neurosci.

[CR155] Tissir F, De-Backer O, Goffinet AM, Lambert de Rouvroit C (2002). Developmental expression profiles of Celsr (Flamingo) genes in the mouse. Mech Dev.

[CR156] Tissir F, Bar I, Jossin Y, De Backer O, Goffinet AM (2005). Protocadherin Celsr3 is crucial in axonal tract development. Nat Neurosci.

[CR157] Torres M, Gomez-Pardo E, Gruss P (1996). Pax2 contributes to inner ear patterning and optic nerve trajectory. Development.

[CR158] Trommsdorff M, Gotthardt M, Hiesberger T, Shelton J, Stockinger W, Nimpf J, Hammer RE, Richardson JA, Herz J (1999). Reeler/Disabled-like disruption of neuronal migration in knockout mice lacking the VLDL receptor and ApoE receptor 2. Cell.

[CR159] Tsui CC, Copeland NG, Gilbert DJ, Jenkins NA, Barnes C, Worley PF (1996). Narp, a novel member of the pentraxin family, promotes neurite outgrowth and is dynamically regulated by neuronal activity. J Neurosci.

[CR160] Wang MM, Reed RR (1993). Molecular cloning of the olfactory neuronal transcription factor Olf-1 by genetic selection in yeast. Nature.

[CR161] Wang LC, Dani J, Godement P, Marcus RC, Mason CA (1995). Crossed and uncrossed retinal axons respond differently to cells of the optic chiasm midline in vitro. Neuron.

[CR162] Wang SW, Mu X, Bowers WJ, Kim DS, Plas DJ, Crair MC, Federoff HJ, Gan L, Klein WH (2002). Brn3b/Brn3c double knockout mice reveal an unsuspected role for Brn3c in retinal ganglion cell axon outgrowth. Development.

[CR163] Wang Y, Thekdi N, Smallwood PM, Macke JP, Nathans J (2002). Frizzled-3 is required for the development of major fiber tracts in the rostral CNS. J Neurosci.

[CR164] White LE, Coppola DM, Fitzpatrick D (2001). The contribution of sensory experience to the maturation of orientation selectivity in ferret visual cortex. Nature.

[CR165] Williams SE, Mann F, Erskine L, Sakurai T, Wei S, Rossi DJ, Gale NW, Holt CE, Mason CA, Henkemeyer M (2003). Ephrin-B2 and EphB1 mediate retinal axon divergence at the optic chiasm. Neuron.

[CR166] Williams SE, Grumet M, Colman DR, Henkemeyer M, Mason CA, Sakurai T (2006). A role for Nr-CAM in the patterning of binocular visual pathways. Neuron.

[CR167] Xiang M, Zhou L, Macke JP, Yoshioka T, Hendry SH, Eddy RL, Shows TB, Nathans J (1995). The Brn-3 family of POU-domain factors: primary structure, binding specificity, and expression in subsets of retinal ganglion cells and somatosensory neurons. J Neurosci.

[CR168] Xu W, Orr-Urtreger A, Nigro F, Gelber S, Sutcliffe CB, Armstrong D, Patrick JW, Role LW, Beaudet AL, De Biasi M (1999). Multiorgan autonomic dysfunction in mice lacking the beta2 and the beta4 subunits of neuronal nicotinic acetylcholine receptors. J Neurosci.

[CR169] Xuan S, Baptista CA, Balas G, Tao W, Soares VC, Lai E (1995). Winged helix transcription factor BF-1 is essential for the development of the cerebral hemispheres. Neuron.

[CR170] Yamaguchi H, Zhou C, Lin SC, Durand B, Tsai SY, Tsai MJ (2004). The nuclear orphan receptor COUP-TFI is important for differentiation of oligodendrocytes. Dev Biol.

[CR171] Zhou C, Qiu Y, Pereira FA, Crair MC, Tsai SY, Tsai MJ (1999). The nuclear orphan receptor COUP-TFI is required for differentiation of subplate neurons and guidance of thalamocortical axons. Neuron.

[CR172] Zinkernagel RM, Doherty PC (1979). MHC-restricted cytotoxic T cells: studies on the biological role of polymorphic major transplantation antigens determining T-cell restriction-specificity, function, and responsiveness. Adv Immunol.

